# Genome-wide analysis and transcript profiling of PSKR gene family members in *Oryza sativa*

**DOI:** 10.1371/journal.pone.0236349

**Published:** 2020-07-23

**Authors:** Preeti Nagar, Ashish Kumar, Muskan Jain, Sumita Kumari, Ananda Mustafiz

**Affiliations:** 1 Plant Molecular Biology Laboratory, Faculty of Life Sciences and Biotechnology, South Asian University, New Delhi, India; 2 School of Biotechnology, Sher-e-Kashmir University of Agricultural Sciences and Technology, Jammu, JK, India; National Institute of Plant Genome Research, INDIA

## Abstract

Peptide signalling is an integral part of cell-to-cell communication which helps to relay the information responsible for coordinating cell proliferation and differentiation. Phytosulfokine Receptor (PSKR) is a transmembrane LRR-RLK family protein with a binding site for small signalling peptide, phytosulfokine (PSK). PSK signalling through PSKR promotes normal growth and development and also plays a role in defense responses. Like other RLKs, these PSKRs might have a role in signal transduction pathways related to abiotic stress responses. Genome-wide analysis of phytosulfokine receptor gene family has led to the identification of fifteen putative members in the *Oryza sativa* genome. The expression analysis of *OsPSKR* genes done using RNA-seq data, showed that these genes were differentially expressed in different tissues and responded specifically to heat, salt, drought and cold stress. Furthermore, the real-time quantitative PCR for fifteen *OsPSKR* genes revealed temporally and spatially regulated gene expression corresponding to salinity and drought stress. Our results provide useful information for a better understanding of *OsPSKR* genes and provide the foundation for additional functional exploration of the rice *PSKR* gene family in development and stress response.

## Introduction

Signal modulation is important for plant developmental process, growth regulation and stress response. This process is mediated by a number of factors including growth regulators and their associated signal transduction pathways. Peptide signalling in plants has been recognized as a central component of cell-to-cell communication responsible for coordinating growth and development [1–3]. Phytosulfokine (PSK) is a Tyr-disulfated pentapeptide with amino acid backbone of YIYTQ that displays hormone-like activities at nanomolar concentrations [4, 5]. It promotes plant growth through elevated cell expansion. Furthermore, its involvement in the response to pathogens has been reported [6–12]. It is synthesised as a proprotein and is post-translationally modified by sulfonylation of the two tyrosine residues by tyrosylprotein sulfotransferase (TPST) in the trans-golgi apparatus [5].

PSK signal is perceived and transduced by a membrane localized receptor protein. The receptor was first isolated as PSK binding protein in microsomal fractions from carrot cell cultures (*Daucus carota*) [13]. Later, its two ortholog genes were identified in *Arabidopsis*, AtPSKR1 and AtPSKR2 [14]. These receptors belong to subgroup X of the LRR-RLKs (leucine-rich repeat receptor-like kinases) family [4, 13]. LRR RLKs are characterized by functional domains like LRRs (leucine rich repeats) with an extrallular ligand binding domain, a single transmembrane domain and a cytoplasmic Kinase Domain (KD) [13, 14]. AtPSKR1 has a predicted extracellular domain with 21 LRRs. PSK binds directly to the 36-amino-acid island domain located between LRR17 and LRR18 of the extracellular receptor domain of AtPSKR1 through its sulphate moieties [13–15]. AtPSKR1 possesses a single helical 21-amino-acid transmembrane domain which is anchored into the plasma membrane followed by a serine/threonine KD with its 12 conserved subdomains [13, 16, 17]. The AtPSKR1-KD has dual guanylate cyclase and kinase catalytic activity. Both the activities are regulated by calcium and this calcium level is sensed by calmodulin (CaM). AtPSKR1- KD also has Ca^2+^/CaM binding site which is essential for its kinase activity [17, 18]. Ligand binding to AtPSKR1 initiates auto and cross phosphorylation cascade. It further stimulates the allosteric changes throughout the receptor resulting in heterodimerization and interaction with other integral membrane proteins. It has been shown that it interacts with BRI1-associated receptor kinase (BAK1)/SERK3 which activate the intracellular receptor kinase domain [19]. Life time fluorescence imaging revealed that AtPSKR1 also interacts with H^+^-ATPases, AHA1 and AHA2 besides BAK1 to form a receptor complex. This complex also associates with the cyclic nucleotide-gated cation channel 17 (CNGC17), although PSKR1 does not directly bind to it [20].

There are various studies which showed the role of PSK- PSKR mediated signalling in normal growth and development. For instance, these receptors are involved in promoting root formation [21], hypocotyl elongation [8], cell growth and differentiation [14], gametogenesis, pollen germination and fertilization [22, 23], somatic embryogenesis and maintenance of procambial cell identity [24]. Further, some studies proposed the involvement of PSK and PSKR in regulating defense response against biotic stresses [12, 25]. PSK acts as a damage-associated molecular pattern and is perceived mainly by AtPSKR1 to provide good protection to plant against pathogen infection [26]. In a recent study, it has been shown that plants overexpressing a rice gene LOC_Os02g41890 (named as OsPSKR1) exhibit enhanced resistance to RS105, a strain of *Xanthomonas oryzae* pv. *oryzicola* (*Xoc*) that causes bacterial leaf streak in rice, and also activates the expression of pathogenesis-related genes involved in the salicylic acid pathway [4, 27].

PSK signalling frequently acts together with phytohormones to coordinate the plant response to external and metabolic stimuli [4]. AtPSKR1 decreases the resistance of Arabidopsis plants to the hemibiotrophic bacterial pathogen *Pseudomonas syringae* but enhances defense against the necrotrophic fungal pathogen *Alternaria brassicicola* [11, 26]. PSK mediates the suppression of resistance to *Pst* DC3000 by inhibiting the salicylic acid (SA)-signalling pathway [11]. In Arabidopsis, PSK is also found to be involved in regulating the copper homeostasis by suppressing ethylene (ET) production [28]. PSK signalling via *Solanum lycopersicum* PSKR1 increases cytosolic [Ca^2+^] and activates auxin-mediated pathways to enhance immunity of tomato plant to *Botrytis cinerea* [25]. However, a comprehensive analysis of its role in response to abiotic stress is not yet reported in any plant system.

There are several evidences which demonstrate that LRR RLKs play a role in a wide variety of signal transduction pathways related to hormone and abiotic stress responses [29, 30]. They are involved in perception, amplification and transmission of environmental stimuli via signalling cascades that finally modulate gene expression, protein activation and cell adjustment [31]. The overproduction of RPK1, an LRR-RLK gene, enhances abiotic stress tolerance in *Arabidopsis* [32]. Likewise, in rice, an LRR-RLK gene OsSIK1, positively regulates plant tolerance to drought and salt stress by involving Ca^2+^ as secondary messenger to trigger stress responses at the nuclear level [33, 34].

Based on subtractive transcriptome profiling of rice seedlings, it has been determined that PSKR is transcriptionally induced in response to salinity stress [35]. The large-scale FGAS wheat EST sequencing project also identified PSKR gene, and found it to be associated with abiotic stress by acting as wheat ice recrystallization inhibition protein homolog and by involvement in signalling cascade [36]. Therefore, PSKR genes in rice may serve as potential candidates in modulating the abiotic stress responses. This study was thus aimed to do genome wide identification and transcript profiling of PSKR genes in *O*. *sativa* to comment on their involvement in development and stress response. Our findings demonstrated that some *OsPSKR* genes might play important role in mediating biotic and abiotic stress signals in rice.

## Materials and methods

### Identification of PSKR gene family members in *O*. *sativa* and phylogenetic analysis

To identify the putative members of PSKR genes in *O*. *sativa*, Hidden Markov Models (HMM) and sequence homology were used. The known PSKR genes in *Arabidopsis* (*At2g02220* and *At5g53890*) were retrieved from TAIR (https://www.arabidopsis.org/) and used to search PSKRs in *O*. *sativa* using HMMER (hmmer.org) against ssp. japonica (IRGSP-1.0). The proteomes were downloaded from Ensembl plants (release 47). In another approach, known PSKR genes from different plants were retrieved from the NCBI protein database and used as query against *O*. *sativa* proteome using BLASTp. The best hits from both approaches were taken and rechecked using NCBI CDD, PFAM (http://pfam.xfam.org/) and SMART (http://smart.embl.de/) for the presence of LRR and kinase domains and only those sequences which had all the domains were considered as *OsPSKR*s. They were numbered in increasing order according to their chromosome number and gene start positions. Chromosomal coordinates of *OsPSKR*s were taken from Ensembl plants biomart (http://plants.ensembl.org/index.html) and were represented on rice chromosomes using MapChart [37]. MCScanX was used to identify segmental and tandem duplicates [38]. One-to-One homologs in *Oryza sativa* subspecies indica (ssp. indica) were taken from ensembl plants biomart. Orthologs of OsPSKRs in other monocots and dicots were taken from NCBI. Phylogenetic relationship between OsPSKRs and its orthologs in monocots and dicots was inferred using neighbour joining method with 1000 bootstrap. Phylogenetic tree was combined with protein domain structure and gene structure using iTOL (https://itol.embl.de/).

### Structural and physicochemical parameters of OsPSKR genes/proteins

Genomic features (UTRs, CDS etc.) of *OsPSKR* genes were retrieved from Ensembl plants (https://plants.ensembl.org/index.html) and protein domain coordinates were determined using SMART (http://smart.embl.de/). These features were represented on *OsPSKR* genes using GSDS 2.0 (http://gsds.cbi.pku.edu.cn/). The isoelectric point (pI) and molecular weight (MW) of OsPSKRs were calculated using ProParam tool of ExPASy (https://web.expasy.org/protparam/). The subcellular localization of OsPSKR proteins was predicted with the online program Plant-PLoc (http://www.csbio.sjtu.edu.cn/bioinf/plant/)

To further confirm the transmembrane helical domains (TMs) in OsPSKR protein, the candidate sequences were scanned using the Phobius (http://phobius.sbc.su.se/) which predicts the transmembrane helices and signal peptide in proteins.

### Multiple sequence alignment of their kinase domains and homology modelling

Multiple sequence alignment of kinase domain of all OsPSKRs with already known PSKRs from *Arabidopsis thaliana* (AtPSKR1 and AtPSKR2) and *Daucus carota* (DcPSKR), was performed using Clustalw (https://www.genome.jp/tools-bin/clustalw) program and visualized using ESPript 3.0 (http://espript.ibcp.fr/ESPript/cgi-bin/ESPript.cgi) [39]. All the OsPSKR members were modelled three-dimensionally using the Phyre2 (http://www.sbg.bio.ic.ac.uk/phyre2/html/) server in the intensive mode [40].

### Gene expression profiling of *OsPSKR* genes and construction of co-expression network

Raw RNA-seq data of ssp. japonica from SRP039045 and PRJNA530826 were retrieved from NCBI SRA which contains RNA-seq of tissue specific expression (root, panicle and leaf) and rice plants affected with drought, heat and salinity across seven time points, respectively. Additionally, TPM values for pre-processed RNA-Seq data from EBI expression atlas (https://www.ebi.ac.uk/gxa/home) were retrieved for expression across eight tissues (reproductive organs) (E-MTAB-2039), *Xanthomonas oryzae* infection (E-GEOD-67588) and cold stress in susceptible and resistant cultivars (E-MTAB-5941). RNA-seq reads from PRJNA523846 (*Pyricularia oryzae* infection), PRJNA628609 (salinity stress) and PRJNA607661 (cold stress) were retrieved from NCBI SRA for ssp. indica.

TPM values were generated from raw reads by mapping them on *Oryza sativa* transcriptome using salmon with default parameters [41]. Ratios of TPM values in treatment and control were log2 transformed and plotted as heatmap. In case of tissues specific expression TPM values were scaled and centered before plotting as heatmap. Log fold changes or TPM values were averaged across replicates before plotting the heatmap.

Co-expressing genes for ssp. japonica were retrieved from Rice Expression Database (http://expression.ic4r.org/) using 0.9 as the absolute cutoff of Pearson’s correlation coefficient. Co-expressing genes in the database are inferred from 284 high quality RNA-seq experiments which includes biotic stress, abiotic stress and tissue specific experiments. A network of these genes was constructed using Cytoscape [42] where edge weight represents correlation strength, thicker edge means higher correlation. Nodes represent individual genes. Edge length is arbitrary and does not have any significance. Orphan nodes were removed from the network.

### Identification of putative cis-acting regulatory elements in *OsPSKR*s

Genomic region 1500 bp upstream of *OsPSKR* genes was retrieved from ensembl plants and *cis*-acting regulatory elements (CAREs) were identified using PLANTcare database (http://bioinformatics.psb.ugent.be/webtools/plantcare/html/) and the data is depicted in the form of heat map using ClustVis (https://biit.cs.ut.ee/clustvis/). Transcription factor targets and gene ontology terms were retrieved from PlantRegMap (http://plantregmap.cbi.pku.edu.cn/). Transcription factor targets at PlantRegMap are determined using CHIP-seq and literature review.

### Plant material, growth conditions and stress treatment

The seedlings of IR64 rice were grown under standard growth conditions in the growth chamber at 28±2°C with a photoperiod of 16 h and humidity of 70–80%. The seeds were sterilized with 1% Bevistin for 20 min and allowed to germinate in hydroponic system. The germinated seeds were then supplied with Yoshida Media [43]. The rice seedlings were allowed to grow for 10 days following which they were exposed to salinity stress (200 mM NaCl dissolved in yoshida media) and drought stress (seedlings removed from hydroponics followed by desiccation on a tissue paper towel) for a time-period of 1 and 24 h whereas untreated seedlings were used as control [35, 44–46].

### Total RNA isolation and expression analysis by real time PCR

Total RNA was isolated from shoot and root tissue of control and stressed rice plants using IRIS kit (Bangalore, Genei) as per the manufacturer’s protocol. RNase free DNase I (Fermentas Life Sciences, USA) enzyme was used to get rid of the genomic DNA contamination in RNA samples. First strand cDNA synthesis was carried out using Maxima first strand cDNA synthesis kit for qPCR-RT (Fermentas Life Sciences, USA). Primers for real-time PCR analysis of *OsPSKR* genes in rice were designed using NCBI primer BLAST for a product length ranging between 70 and 120 bp (list of primers given in S1 Table). Rice *β-tubulin* gene was used as reference gene for the normalization of real-time data. The PCR mixture contained 2.5 μl first strand cDNA (10 times diluted), 5 μl of 2X SYBR green PCR master mix (Fermentas Life Sciences, USA), and 2 μM of each gene-specific primer in a final volume of 10 μl. Negative template controls (NTC) were also performed for each of the primer pair. The real-time PCRs were performed employing ViiA7^TM^ real-time PCR machine (Applied Biosystems, USA). All reactions were performed under the following conditions: 10 min at 95°C, and 40 cycles of 15 s at 95°C, 30 s at 60°C and melt curve with single reaction cycle with following conditions 95°C for 15 s, 60°C for 1 min and dissociation at 95°C for 15 s. Three biological replicates were analyzed for each sample. The relative expression ratio was calculated using delta Ct value method [47].

## Results

### Identification of *OsPSKR*s, their chromosomal distribution and gene duplication

We have identified fifteen *OsPSKR* genes in *Oryza sativa* using HMM profiles and sequence homology-based searches. Table 1 gives a list of *OsPSKR* genes identified in *O*. *sativa* ssp. japonica along with their genomic and protein features. *OsPSKR*1 (4288 bp) was the longest and *OsPSKR*13 (3198 bp) was the smallest gene, however, all OsPSKR proteins were of similar length, their sizes ranged from 1013 to 1067 amino acids. Most of the OsPSKR proteins have relatively low isoelectric points (pI < 7), except for OsPSKR4, which has a pI of 7.06.

**Table 1:** List of PSKR gene family members present in *Oryza sativa* along with their localization coordinates, gene and protein details.

No.	Gene Locus	Name	Chr	Coordinates	Gene (bp)	CDS (bp)	Protein (aa)	MW (KDa)	pI	Plant-Ploc prediction
1	LOC_Os02g02490.1	OsPSKR1	2	884,018–888,245 forward strand.	4228	3183	1061	111.6	5.7	plas
2	LOC_Os02g05910.1	OsPSKR2	2	2,933,894–2,937,379 reverse strand.	3486	3156	1052	115.4	6.0	plas
3	LOC_Os02g05920.1	OsPSKR3	2	2,938,046–2,941,648 reverse strand.	3603	3153	1051	115.7	6.8	plas
4	LOC_Os02g05930.1	OsPSKR4	2	2,945,293–2,949,306 reverse strand.	4014	3192	1064	116.2	7.1	plas
5	LOC_Os02g05940.1	OsPSKR5	2	2,952,131–2,955,766 reverse strand.	3636	3150	1050	115.1	5.9	plas
6	LOC_Os02g05950.1	OsPSKR6	2	2,958,769–2,962,177 reverse strand.	3409	3144	1048	115.1	5.6	plas
7	LOC_Os02g05960.1	OsPSKR7	2	2,965,240–2,968,658 reverse strand.	3419	3156	1052	115.5	6.2	plas
8	LOC_Os02g05970.1	OsPSKR8	2	2,971,987–2,975,382 reverse strand.	3396	3141	1047	114.7	5.5	plas
9	LOC_Os02g05980.1	OsPSKR9	2	2,978,855–2,982,402 reverse strand.	3540	3150	1050	115.6	5.9	plas
10	LOC_Os02g418901.1	OsPSKR10	2	25,176,090–25,179,701 reverse strand.	3612	3159	1053	114.9	6.3	plas
11	LOC_Os04g57630.1	OsPSKR11	4	34,298,775–34,302,683 reverse strand.	3909	3039	1013	109.7	5.9	plas
12	LOC_Os06g47650.1	OsPSKR12	6	28,853,371–28,856,936 forward strand.	3566	3201	1067	116.9	6.3	plas
13	LOC_Os06g47740.1	OsPSKR13	6	28,888,959–28,892,150 reverse strand.	3192	3192	1064	114.3	5.5	plas
14	LOC_Os06g47750.1	OsPSKR14	6	28,898,441–28,901,943 reverse strand.	3503	3201	1067	114.6	5.9	plas
15	LOC_Os07g01710.1	OsPSKR15	7	424,870–428,523 forward strand.	3654	3108	1036	111.6	6.2	plas

MW: molecular weight, pI: isoelectric point, plas: plasma membrane.

All *OsPSKR* genes from *O*. *sativa* ssp. japonica were mapped on the rice chromosomes (S1 Fig). *OsPSKR* genes were present only on four chromosomes, 2, 4, 6 and 7. Chromosome 2 had the highest density with ten *OsPSKR* genes followed by chromosome 6 with three while Chromosome 4 and 7 each had one *OsPSKR* gene. Twelve out of fifteen genes were present on the minus strand. Gene duplication is among the primary mechanisms of gene family growth [23]. Tandem and segmental duplications in *OsPSKR* genes were analyzed and four tandemly duplicated gene pairs and one segmental duplication event were found (S1 Fig).

### Phylogenetic relationship and analysis of structural features of PSKR genes/proteins in *O*. *sativa*

**Fig 1:**
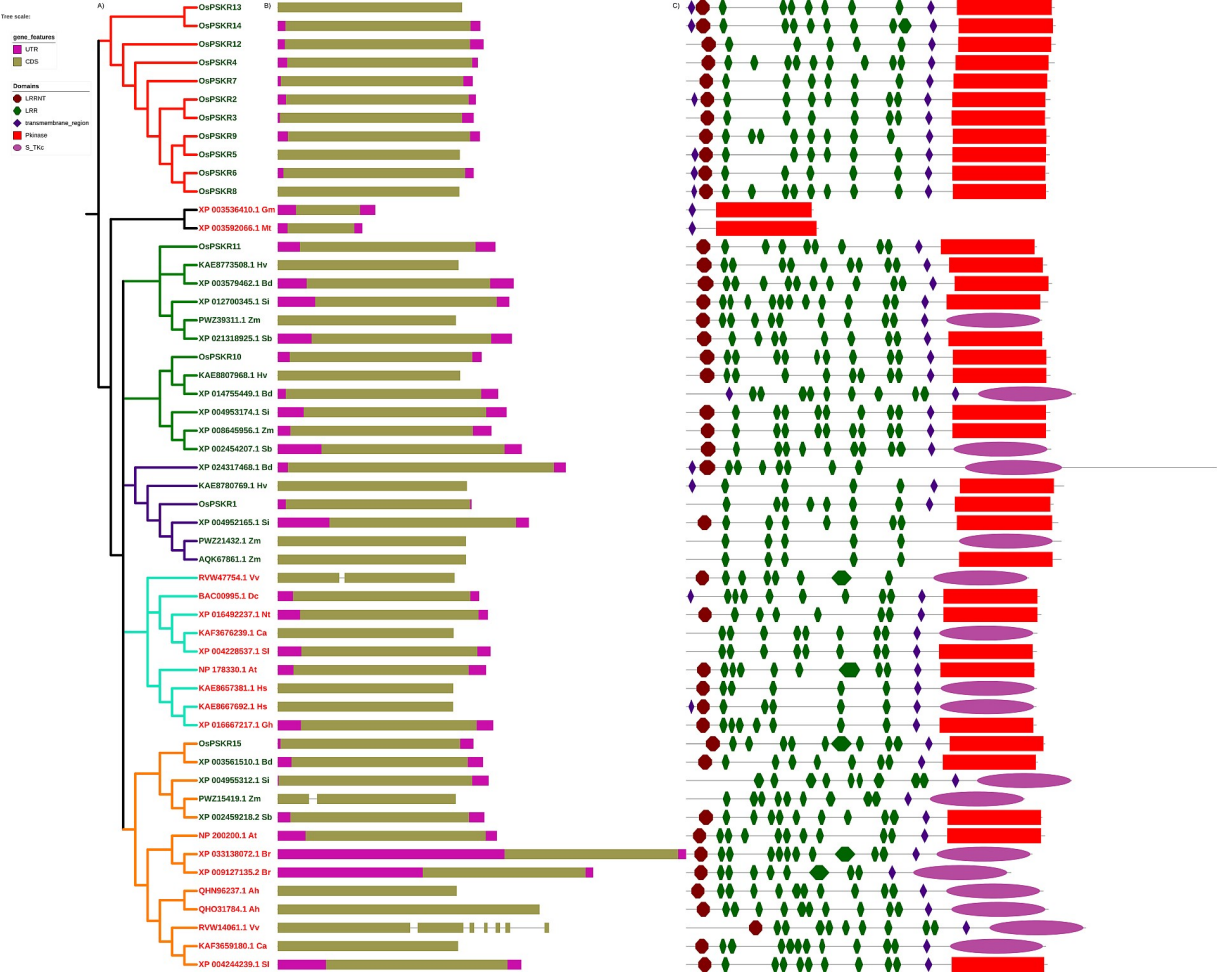
Phylogenetic relationship, gene and domain structure of PSKRs. Neighbour joining tree representing the phylogenetic relationship between full length OsPSKRs and their orthologs in both monocots and dicots. Node labels are colored green for monocots and red for dicots. Node labels are suffixed by a two letter code to indicate the species name. Hv: *Hordeum vulgare*; Bd: *Brachypodium distachyon*; *Si*: *Setaria italica*; Zm: *Zea mays*; Sb: *Sorghum bicolor*; Vv: *Vitis vinifera*; Dc: *Daucus carota*; Nt: *Nicotiana tabacum*; Ca: *Capsicum annuum*; Gm: *Glycine max*; Mt: *Medicago truncatula*; Sl: *Solanum lycopersicum*; At: *Arabidopsis thaliana*; Hs: *Hibiscus syriacus*; Gh: *Gossypium hirsutum*; Ah: *Arachis hypogea*; Br: *Brasicca rapa*. B) Exon/intron structure of PSKR genes. C) Domain organization of PSKR proteins.

A phylogenetic tree was constructed to see the relation of OsPSKRs with orthologs in both monocots and dicots (Fig 1A). PSKRs from monocots and dicots clustered separately. Due to the high number of tandem duplicates in OsPSKR gene family, most of the OsPSKRs form a cluster which was separate from the orthologs. This was expected since the tandem duplicates has more sequence similarity between themselves than the orthologs. *OsPSKR*1, 10, 11 and 15 which were not part of any tandem duplicate array cluster with their monocot orthologs. Gene structure along with the protein domains were also analyzed for all the *PSKR* genes (Fig 1). All *PSKR* genes were intronless having single CDS region with three exceptions RVW14061.1 (*Vitis vinifera*) had six introns whereas RVW47751.1 (*Vitis vinefera*) and PWZ15419.1 (*Zea mays*) had one intron each (Fig 1B). Usually, recently evolved genes or pseudogenes originated from retro-transposition are intronless, they arise by copy-paste type mechanism [48, 49]. OsPSKR proteins were flanked by Pkinase domain (PF00069) on the C-terminal and LRRNT domain (PF08263), Cysteine rich regions, on the N-terminal except. OsPSKR1, OsPSKR7 and OsPSKR8 lack LRRNT domain in its N-terminal (Fig 1C). Few orthologs were found to have S_Tkc (Ser/Thr kinase) domain instead of Pkinase. The transmembrane region was present in all OsPSKR members and tandem LRR repeats were interspersed in between the LRRNT domain and the transmembrane region.

Both monocot and dicot PSKRs can be divided into two groups each based on the number of LRR domains. These families are represented using different colored branches. Orange clade consisting of dicots has higher density of LRR domains than the blue clade of dicots. Similarly, green clade of monocots has higher LRR density compared to the purple clade of monocots.

### *In silico* prediction of subcellular localization, protein topology and homology modelling of PSKR proteins in rice

Subcellular localization analysis indicated that all OsPSKRs are predominately present on the plasma membrane like the already characterized PSKRs from Arabidopsis [20] (Table 1). Further the amino acid sequences of OsPSKRs were scanned using Phobius (http://phobius.sbc.su.se/) which predicted that all the OsPSKRs have one signal peptide at N-terminal and one transmembrane region with highest probability along with defined cytoplasmic and non-cytoplasmic regions (S2 Fig), suggesting that all members of PSKR family in rice are single pass transmembrane proteins.

**Fig 2:**
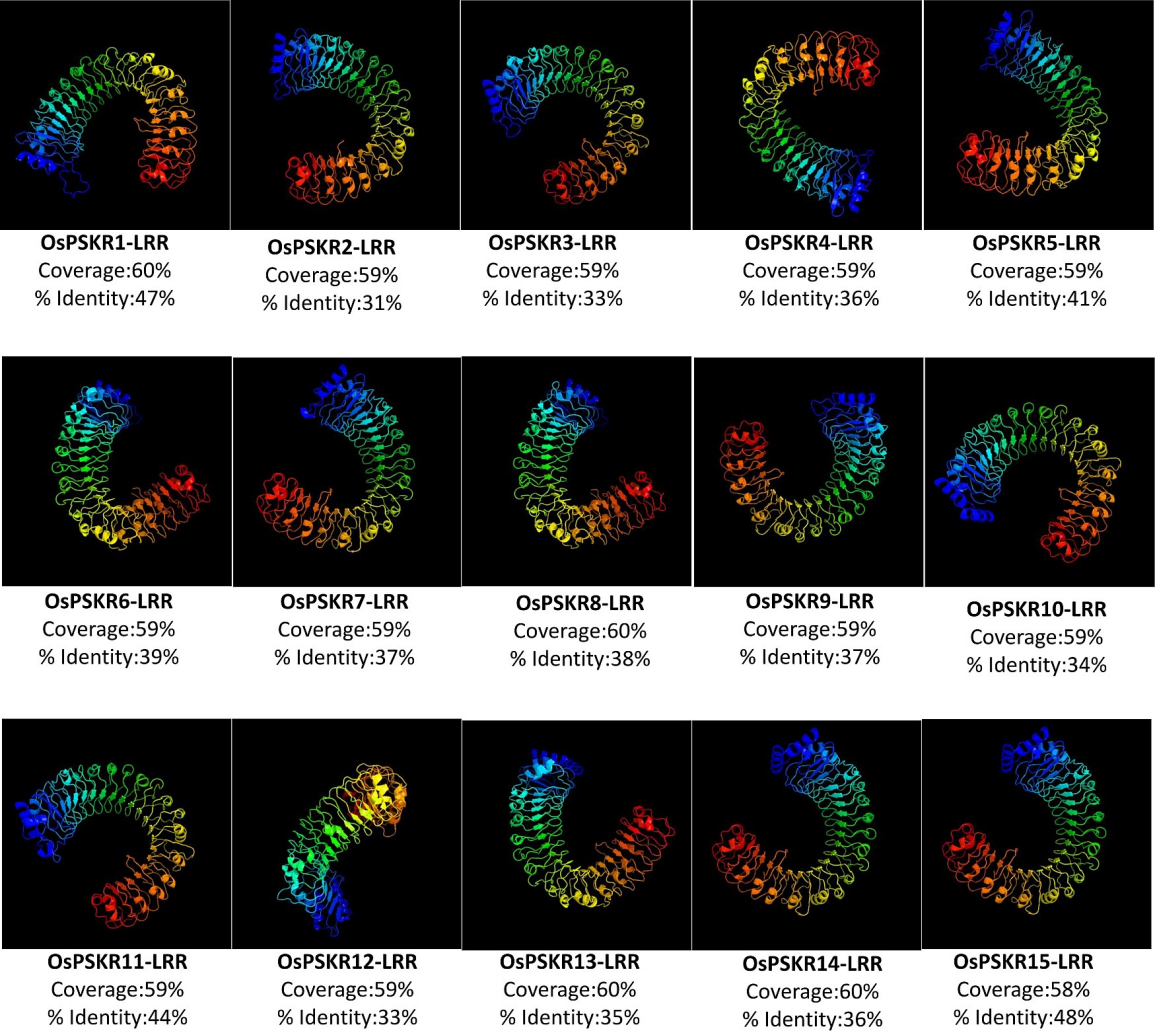
Predicted 3D models of LRR domain of rice PSKR proteins. Models were generated by using Phyre2 server. Models were visualized by rainbow color from N to C terminus. C4z62A of PSKR1 (from *Arabidopsis thaliana*) was used as template in the modelling of ectodomain of OsPSKR proteins. The alignment coverage and % identity of the predicted model with the template has been shown below each model.

All fifteen rice PSKR family members were three-dimension modelled using Phyre2 (Protein Homology/analogY Recognition Engine) server (http://www.sbg.bio.ic.ac.uk/phyre2/) (S3 Fig). Predicted models were based on the reported 10 templates (c4y93A, c6s6qB, c2j0kB, c1oplA, c4mnaA, c4xi2A, c1y57A, c5gr8A, c4mn8A and c2fo0A) to heuristically maximize the alignment coverage, percentage identity, and confidence score for the tested sequences. About 87–93% residues (with 90% coverage score) of OsPSKRs were aligned with corresponding model sequences, suggesting that the OsPSKR structure predictions are highly reliable. In OsPSKR proteins, the main predicted secondary structures were α-helix (24–26%) and β-strands (20–22%). Transmembrane (TM) helices were detected in all the OsPSKRs and occupied for 2–4% (S2 Table). In addition, in order to clarify the similarity or difference of the generated models, the superposition structures were used to calculate the percentage of structural coverage. In this study, c4z62A template representing ectodomain (LRR) of phytosulfokine receptor 1 from Arabidopsis was used to predict the tertiary structure of all the identified OsPSKRs with 100% confidence score (Fig 2). All the OsPSKR-LRRs aligned with reported template (with 58–60% alignment coverage) showed 31–48% identity. OsPSKR15-LRR showed 48% identity while OsPSKR2-LRR had minimum identity of 31% with the reported model. The amino acid residues of PSKR that may interact with PSK as well as co-receptor (SERK) were also marked in respective OsPSKR-LRR region based on solved crystal structure data of PSKR from *Daucus carota* (DcPSKR) and *Arabidopsis thaliana* (AtPSKR1/2) (PDB ID: 4Z5W) (available at http://www.rcsb.org/) and the published study [19] (S4 Fig). 3D modelling results revealed that these rice PSKRs showed tertiary structure similarity, implying that rice PSKRs may have evolved from the same ancestor sequence and/or under purification selection force to keep stabilization during long-term acclimation after the initially divergence.

### Multiple sequence alignment and organization of kinase domain of OsPSKRs

**Fig 3:**
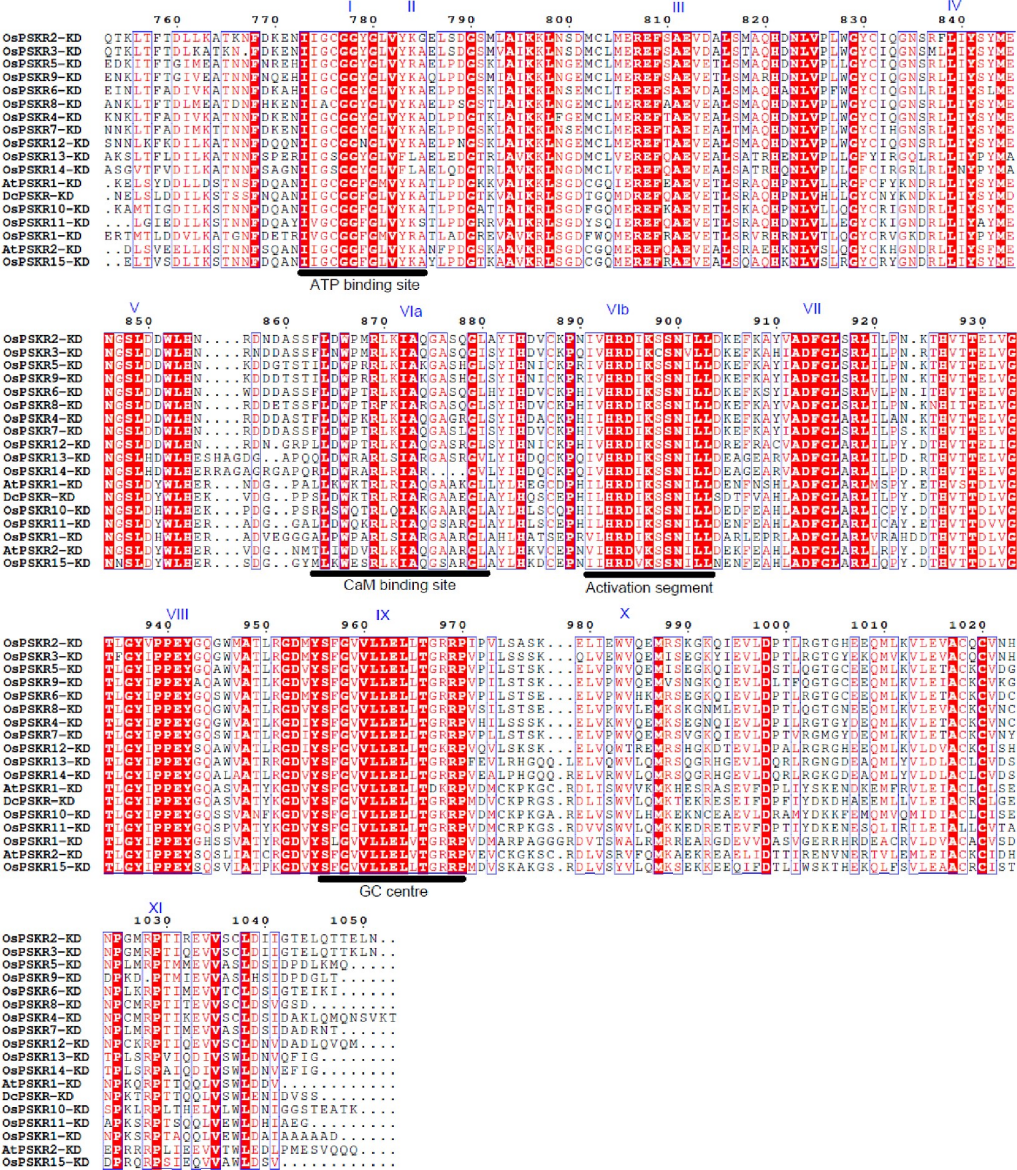
Sequence alignment of kinase domain of the PSKRs of *Arabidopsis thaliana*, *Daucus carota* and *Oryza sativa*. Sequence alignment of the kinase domain of OsPSKRs with carrot DcPSKR and *Arabidopsis* AtPSKR1/2. Conserved and similar residues are boxed with red ground and red font, respectively. Alignments showing the conserved twelve signature subdomains of the PSKR family (marked in romans above the sequences). ATP binding site, CaM (calmodulin) binding site, activation segment and guanylate cyclase centre also indicated in the alignment (black underlined).

In previous studies, PSKRs from *Arabidopsis thaliana* (AtPSKR1 and AtPSKR2) and *Daucus carota* (DcPSKR) were already characterized based on the presence of characteristic features of serine/threonine protein kinases [19, 50]. The amino acid sequence of the kinase domain of all the PSKRs of *O*. *sativa* were aligned with that of the characterized PSKRs. Sequence alignment indicated that all of the OsPSKRs proteins contained highly conserved regions, spanning approximately 300 amino acids near the N-terminal region that were composed of 11 characteristic domains (I–XI) in Fig 3. They all have highly conserved RD-motif in subdomain VIb. The other conserved sites like ATP binding site, calmodulin-binding site, activation segment and guanylate cyclase centre have also been identified in all the OsPSKRs.

Membrane helices prediction for the A. Nodules Hat-E transporters (predicted via TMHMM; http://www.cbs.dtu.dk/services/TMHMM/) and long C-terminal tails. The Hat (A) and Hated (C) contain 12 helices, while Hatch (B) and HxtE (D) contain 10 helices.

### Expression profiling of *OsPSKR* genes using RNAseq data

**Fig 4:**
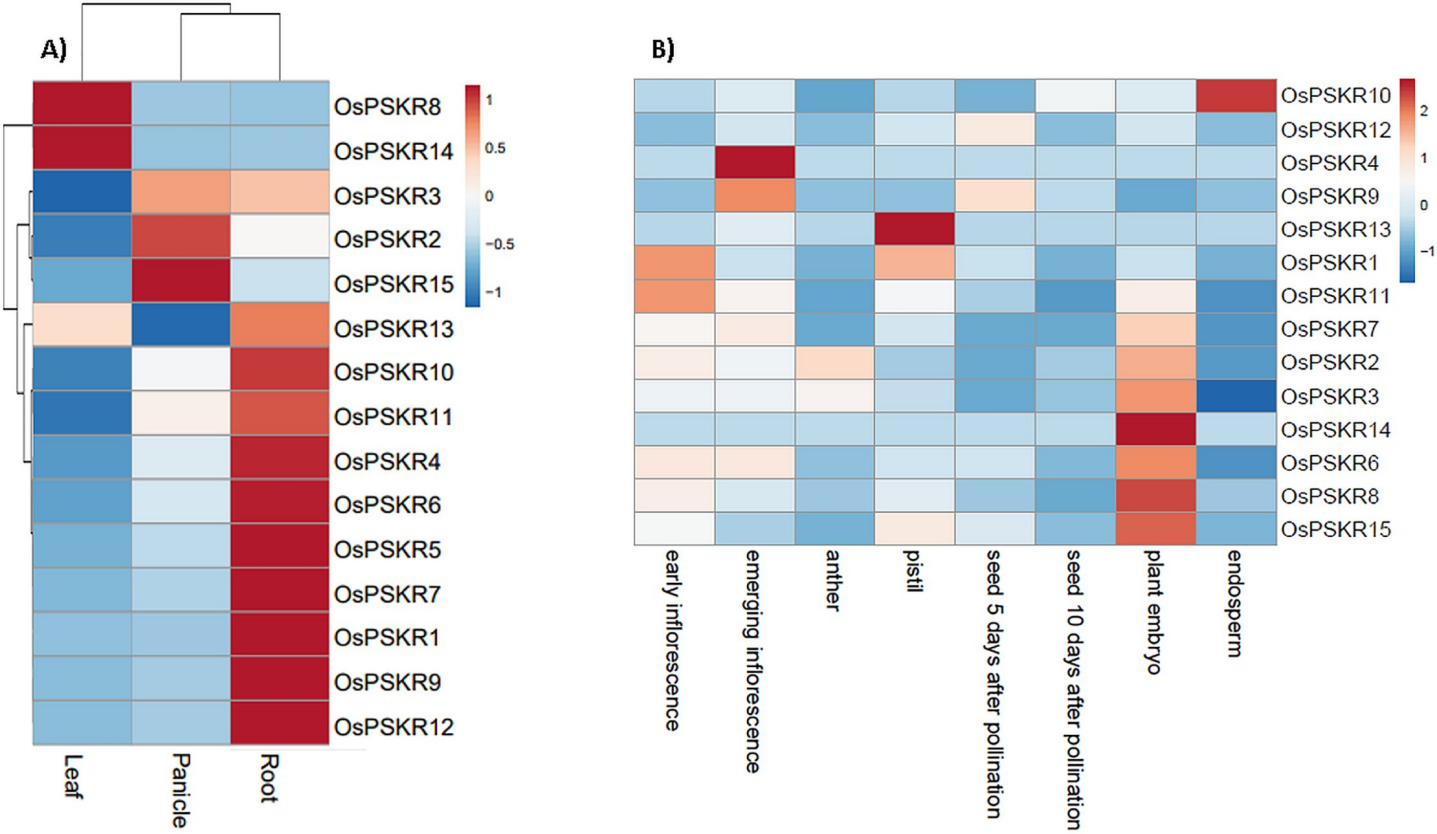
Heatmap showing the expression pattern of 15 *OsPSKR* genes in different tissues. A) Three different tissues used for expression analysis include leaf, panicle and root. B) Expression pattern across flowering and seed tissues. The expression values were scaled and centered before creating the heatmap. Red indicates high concentrations, whereas low relative concentrations are deep blue.

**Fig 5:**
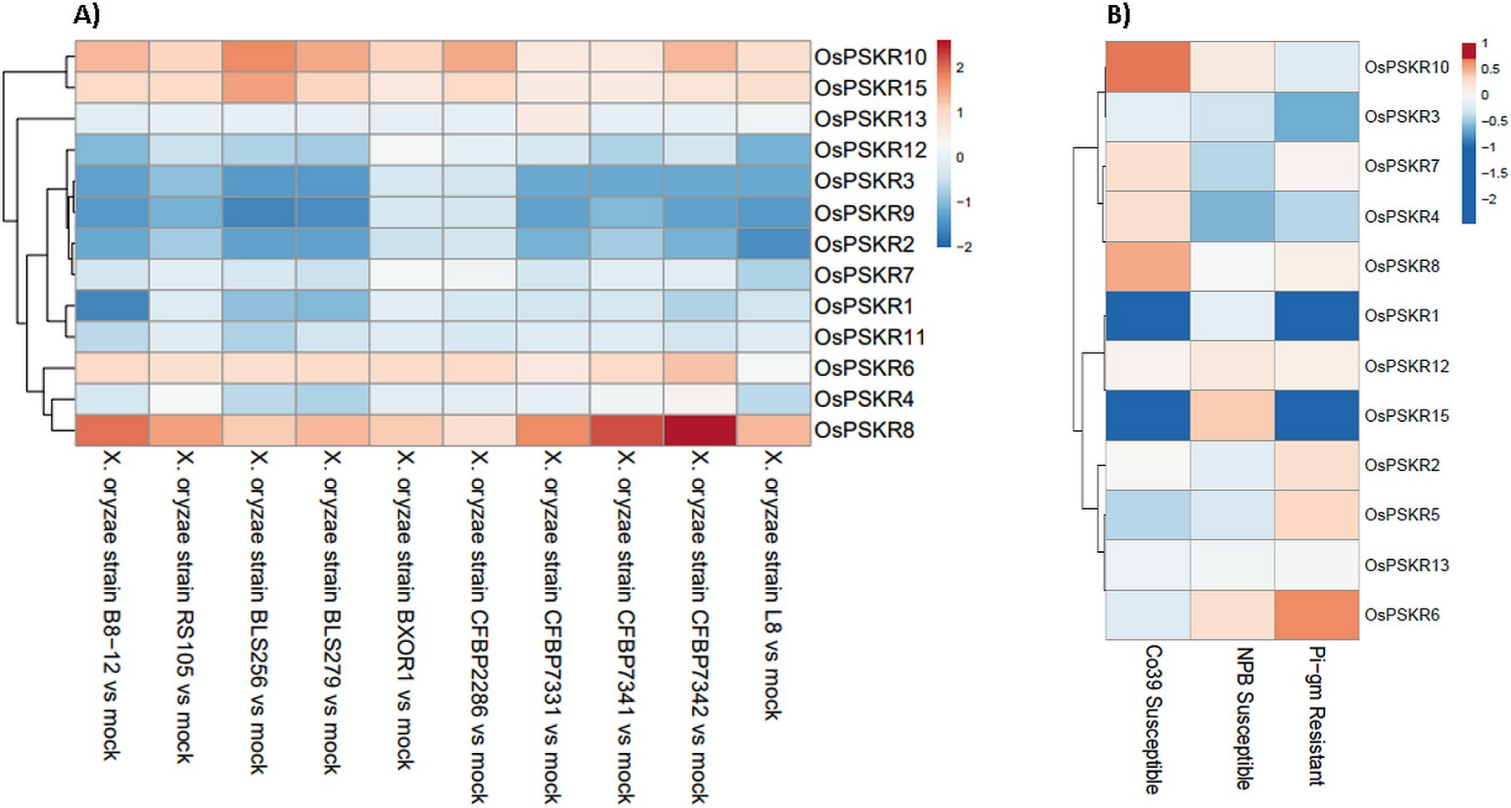
Heatmap showing the expression pattern of *OsPSKR* genes under biotic stress conditions. A) Expression pattern profile of *PSKR* genes of *Oryza sativa* ssp. japonica cv. Nipponbare inoculated with 10 geographically diverse strains of *Xanthomonas oryzae* pv. *Oryzicola* (*Xoc*) for 48 h. B) Expression pattern profile of *PSKR* genes of *Oryza sativa* ssp. indica in susceptible (CO39 and NPB) and resistant (Pi_gm) rice cultivars which were spray inoculated with *Pyricularia oryzae* spore and incubated for 12 h. The TPM values were scaled and centered before plotting the heatmap.

**Fig 6:**
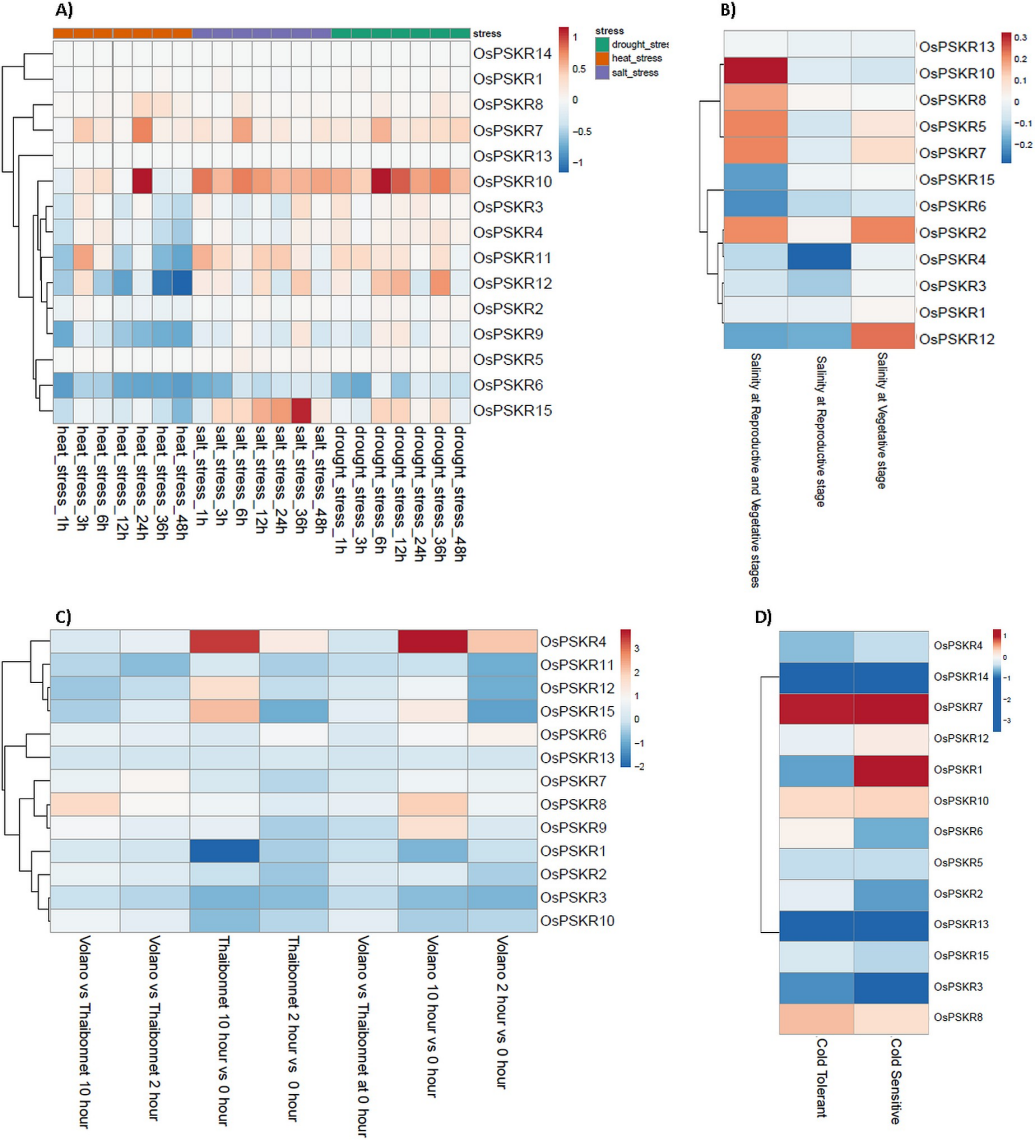
Heatmap showing the expression profiles of *OsPSKR* genes under different abiotic stress conditions. A) Expression pattern profile of *PSKR* genes of *Oryza sativa* ssp. japonica under heat, salinity and drought condition at different time points. B) Expression pattern profile of *PSKR* genes of *Oryza sativa* ssp. indica in reproductive, vegetative and both tissue under salinity stress. C) Expression pattern profile of *PSKR* genes of *Oryza sativa* ssp. japonica in both cold sensitive (Thaibonnet) and tolerant (Volano) under cold condition for 2 h and 10 h time duration. D) Expression pattern profile of *PSKR* genes of *Oryza sativa* ssp. indica in cold sensitive and tolerant cultivars under cold stress. The TPM values were scaled and centered before plotting the heatmap.

RNA transcript profiling is an important strategy to study the expression of a large number of genes. To understand the expression pattern of *OsPSKRs* in different tissues and in response to different stresses (both biotic and abiotic), RNA-seq data was used to retrieve the expression profiles of *OsPSKR* genes (Figs 4–6). It was observed that most of the *OsPSKR* genes were expressed in root tissue with exception of *OsPSKR*2, 8, 14 and 15 (Fig 4A). *OsPSKR*2 and 15 were exclusively expressed in the panicle while *OsPSKR*8 and 14 in the leaf tissue. *OsPSKR*3 showed expression in both panicle and root tissue. Further, the expression profiles for different parts of flower and seeds were also explored (Fig 4B). Multiple *OsPSKR*s (*OsPSKR*2, 3, 6, 7, 8, 14 and 15) show upregulation in the plant embryo tissue where *OsPSKR*14 showed the highest upregulation, which suggested that they probably have diverse functions in seed development. Interestingly, all of these *OsPSKR*s show downregulation in endosperm tissue where OsPSKR3 showed the maximum downregulation. *OsPSKR*10 which does not show any change in embryo tissue showed upregulation in the endosperm tissue. Upregulation was also observed in other parts of flower such as pistil (*OsPSKR*1, 13 and 15) and emerging inflorescence (*OsPSKR*4 and 9).

To better understand the putative role of *OsPSKR*s in response to different abiotic and biotic stresses, the RNA-seq based expression profiles of all fifteen PSKRs were analyzed for both ssp. japonica and indica. As seen in the heatmap (Fig 5A), RNA-seq data of leaves of *Oryza sativa* ssp. japonica showed the upregulation of *OsPSKR*6, *OsPSKR*8, *OsPSKR*10 and *OsPSKR*15 under disease condition caused by almost all strains of *Xoc*. Moreover, *OsPSKR*8 showed the most significant change (increase) in its expression on exposure to *Xoc* strains CFBP7331, CFBP7341, CFBP7342 and B8-12 while other members showed downregulation in their expression upon infection. This results depicted the involvement of OsPSKRs in regulating stress response in rice plant against *Xoc* as has been reported previously [27].

In case of ssp. indica, expression profiles of OsPSKRs were used for both susceptible (CO39 and NPB) and resistant (Pi_gm) rice cultivars (Fig 5B). The results depicted that *OsPSKR*2, *OsPSKR*5 and *OsPSKR*6 gets upregulated in the resistant rice plant on infection with *P*. *oryzae*. It was interesting to note that *OsPSKR*2 and *OsPSKR*5 were only expressed in resistant (Pi_gm) while the expression of *OsPSKR*8 and *OsPSKR*10 was increased in susceptible cultivar. There was increase in transcript level of *OsPSKR*12 in both susceptible as well as resistant cultivars. It was also observed that *OsPSKR*1 and *OsPSKR*15 were downregulated in Pi_gm and CO39, while their expression comparatively increased in NPB.

The expression patterns of different *OsPSKR*s for ssp. japonica and indica under abiotic stress were also analyzed. According to the heat map (Fig 6A), in ssp. japonica, there was increase in expression of *OsPSKR*10 in response to heat, salinity as well as drought stress at most of the time points. *OsPSKR*1, *OsPSKR*13 and *OsPSKR*14 did not show any change (up/down regulation) in any abiotic stress condition. Under heat stress, it was observed that *OsPSKR*7 and 10 transcript level increased at 24 h. However, some of the genes got downregulated like *OsPSKR*6, *OsPSKR*9 *and OsPSKR*12. There was no significant change in expression of other OsPSKRs under heat stress. *OsPSKR*7, 11, 12 and 15 gene expression increased under salinity and drought conditions. Moreover, the expression of *OsPSKR*15 increases in a gradient like manner from 1 h to 36 h in salt stress. RNA-seq data of ssp. indica was available only for salinity stress. In the experiment, salinity stress was given at either vegetative or reproductive stage or both stages. As pointed out in the Fig 6B, *OsPSKR*2 and *OsPSKR*12 were upregulated in vegetative tissue sample, while there was no significant change in expression in the plant at reproductive stage. Moreover, when rice plants were exposed to salt stress in two events as first shock event at vegetative and other at reproductive stage, the expression of *OsPSKR*2, *OsPSKR*5, *OsPSKR*7, *OsPSKR*8 and *OsPSKR*10 got enhanced with *OsPSKR*10 showing the most significant increase.

The expression pattern of *OsPSKR* genes were analyzed from RNA-seq data of Thaibonnet (cold sensitive and Volano (cold tolerant) rice cultivars of ssp. japonica which were given cold stress for 0, 2 and 10 h (Fig 6C). Heatmap showed that the expression of *OsPSKR*4, *OsPSKR*12 and *OsPSKR*15 increased from early (2 h) to late time point (10 h) in both the cultivars. *OsPSKR*4 showed both early as well as late-term responses with more significant upregulation at late duration. In case of Volano, the resistant one, it was observed that besides the above three *OsPSKR*s, the expression of *OsPSKR*8 and *OsPSKR*9 also increased at the late time point. As observed in the heatmap, the results indicated that most of the *OsPSKR*s were downregulated at early time point with slight change in their expression with increase in time. In ssp. indica, *OsPSKR*4 and *OsPSKR*15 were observed to be downregulated unlike ssp. japonica (Fig 6D). *OsPSKR*7, *OsPSKR*8 and *OsPSKR*10 were upregulated in both cold resistant and sensitive cultivars with *OsPSKR*7 showing the most significant increase in its expression. The transcript level of *OsPSKR*1 increased in cold sensitive cultivar while it got downregulated in other resistant cultivar.

On comparing the expression profiles of *OsPSKR*s in both subspecies, we observed that *OsPSKR*6, 8, 10 and 15 were more responsive to both bacterial and fungal infection. Similarly, under saline conditions, *OsPSKR*7, 10 and 12 were upregulated in ssp. japonica as well as indica while *OsPSKR*15 showed significant change in its expression in ssp. japonica but not in ssp. indica. More interestingly, there was difference in expression pattern of *OsPSKR*s under cold stress in both subspecies, as *OsPSKR*4 and 15 were abundant in ssp. japonica while their expression decreased in ssp. indica. As seen in Fig 6D, the transcript level of *OsPSKR*1 and 7 increased in ssp. indica whereas there was no significant change in their expression in another ssp. under cold condition. But *OsPSKR*8 and 12 showed similar response in both ssp. under same stress. Although some *OsPSKR*s show conformity in expression pattern in both the rice ssps. but there is a distinct genotype specific stress regulated expression particularly under cold stress. Further, study in the cold stress response of specific varieties chosen for RNA-Seq analysis will shed more light on the precise role of OsPSKRs in these genotypes.

### Co-expression network analysis

**Fig 7:**
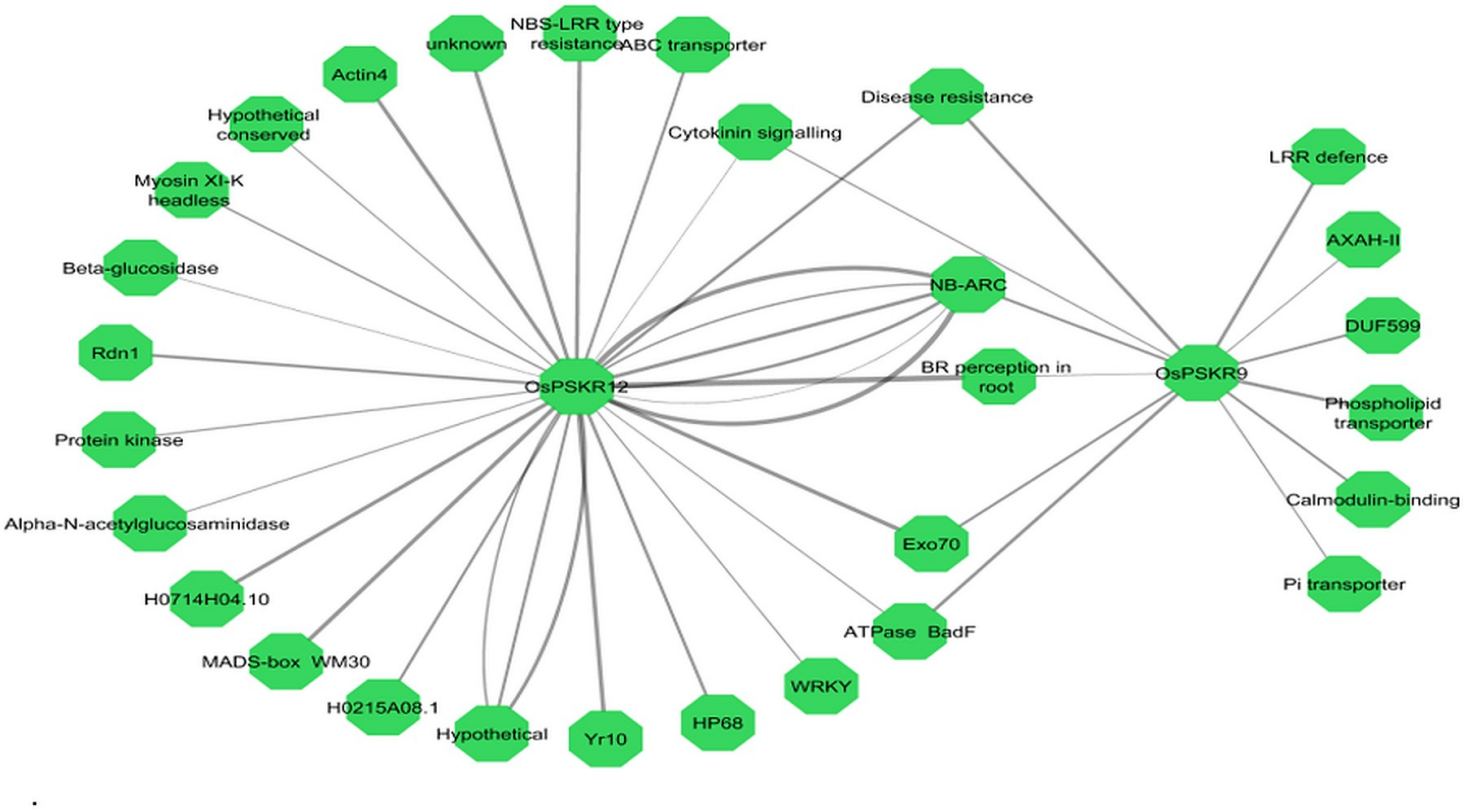
Co-expression network of OsPSKR genes with themselves and with other genes involved in stress/defense responses and development stage related responses using absolute PCC ≤ 0.9. Nodes represent genes, edge weight represents correlations, and multiple edges from single origin represents multiple members of the same gene family. Orphan nodes were excluded from the network.

Another way of functionally characterizing genes is to correlate expression of different genes across different conditions. Clusters of co-expressing genes have a strong probability of being involved in similar functions or regulated by similar mechanisms. Using an absolute cutoff value of Pearson’s correlation coefficient 0.9, two hubs *OsPSKR12* and *OsPSKR*9 were identified (Fig 7).

These are co-expressed with multiple genes involved in stress and defense responses, development stage related responses, trafficking, hormone responses, other kinases and some genes with unknown functions. *OsPSKR*12 has a very strong correlation with R-genes encoding proteins like NBS-LRR (consist of central Nucleotide Binding Site (NBS) and carboxyl/C-terminal Leucine Rich Repeat (LRR) domain), NB-ARC (the central NBS domain) and Yr10 (Stripe rust resistance). NBS-LRR proteins are mainly intracellular receptors that recognize pathogen effector target proteins directly or indirectly in host and activates the defense signal transductions to induce NLR-triggered immunity [51, 52]. Other co-expressing genes include Exo70, WRKY, MADS-box and ABC transporters. Exo70, a component of the exocyst complex, is involved in vesicle trafficking like exocytosis and play crucial role in plant immunity and pollen growth and maturation [53–56]. While WRKY and MADS-box are the most important class of transcription factors involved in abiotic stress response [57–59]. ABC transporters of plants are engaged in numerous functions, including secondary metabolite transport, phytohormone transport, heavy metal detoxification, and also contributes to salinity and drought resistance in plant [60, 61]. *OsPSKR*9 is also correlated with NB-ARC in addition to phospholipid transporter, calmodulin-binding and phosphate transporter genes, suggesting its involvement in phospholipid-based signalling, Ca^2+^ signalling and maintaining the cytosolic Pi (inorganic phosphate) homeostasis in plants, respectively. *OsPSKR*9 and *OsPSKR*12 both are correlated with gene involved in BR (brassinosteroid) perception in root and it has already been reported in *Arabidopsis* that PSK signalling act together with brassinosteroid signalling to modulate plant development and adaptation to stress [9, 24, 62]. The above results adds to the hypothesis that *OsPSKR*s are involved in multitude of stress responses and developmental process especially in roots.

### Identification and functional classification of putative cis-acting regulatory elements present in upstream region of *OsPSKR* genes and transcription factor enrichment

**Fig 8:**
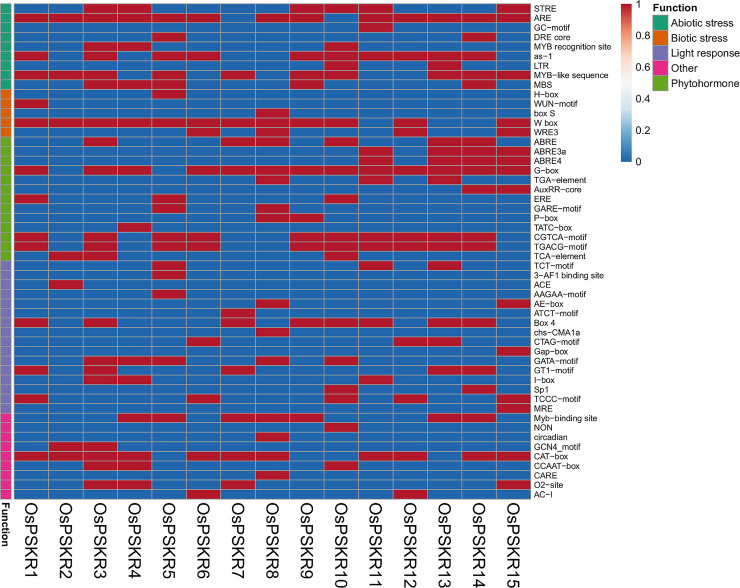
Schematic diagram depicting transcription factor binding sites in *OsPSKR* genes. Heatmap summary of different promoter motifs present in all the *OsPSKR*s. The presence and absence of promoter motifs in the *OsPSKR* family along with their generalized function are shown as a binary heatmap here. Blue color represents absence (zero) and red color represents presence (one). Motif functions are divided into broad categories and they are represented on the left side of the heatmap.

To investigate the cellular mechanism behind the altered expression of *OsPSKR* genes in response to various developmental stages and stresses, the 1500 bp upstream region of the *OsPSKR* genes was analyzed *in silico* to identify the presence of cis-regulatory elements. We identified 51 unique CAREs. Fig 8 represents a heatmap indicating the presence or absence of these motifs in promoter region of each *OsPSKR* gene. This analysis identified several CAREs which play important roles to modulate the molecular switches of dynamic transcriptional regulation. These CAREs are broadly classified into five categories depending upon their response to various factors including hormones, light, abiotic stress, biotic stress and others developmental cues.

Various abiotic stress related motifs were present in promoter region of different *OsPSKR*s like STRE (*OsPSKR*3, 4, 9, 10, 11and 15), AREs (all *OsPSKR*s except *OsPSKR*7 and 10), GC motif (*OsPSKR*11), DRE core (*OsPSKR*5 and 14), MYB-recognition site (*OsPSKR*3, 4 and 10), asI (all *OsPSKR*s except *OsPSKR*2, 4, 7, 8 and 15), LTR (*OsPSKR*10 and 13), MYB like sequences (all *OsPSKR*s except 4, 6, 8, 11,12) and MBS (*OsPSKR*3, 4, 5, 9 and 14).

Some elements responsible for biotic stress signalling such as H-box (*OsPSKR*5), WUN (*OsPSKR*1), Box S (*OsPSKR*8), W-box (all *OsPSKR*s except *OsPSKR*11, 13 and 14) and WRE3 (*OsPSKR*6, 8, 12 and 15) are present in *OsPSKR*s.

Motifs involved in hormonal regulation were found to be second largest in number after light responsive motifs. Few motifs such as ABRE (*OsPSKR*3, 7, 8, 10, 13 and 14), ABRE3a (*OsPSKR*11, 13, 14 and 15) and ABRE 4 (*OsPSKR*11, 13, 14 and 15) are involved in abscisic acid signalling. CGTCA and TGACG, both motifs are present in *OsPSKR*1, 3, 5, 6, 9, 10, 11, 12, 13, 14 and 15 and are found to be responsive to methyl-jasmonate. Auxin responsive elements, AuxRR-core and TGA- element were present in *OsPSKR*14 & 15, and *OsPSKR*8, 11 & 13 respectively. GARE (*OsPSKR*5 and 8) and TATC box (*OsPSKR*4) are cis-acting regulatory elements involved in gibberellin responsiveness. Other motifs like ethylene-responsive ERE (*OsPSKR*5 and 10) and salicylic acid responsive TCA element (*OsPSKR*2, 3 and 10) were also present. The results suggest that OsPSKRs are regulated by different hormones depending on the environmental/developmental cues.

The largest category of cis-elements was associated with light stress response. It includes motifs like TCT motif (*OsPSKR*5, 11 and 13), AF-1 (*OsPSKR*5), ACE (*OsPSKR*2), AAGAA motif (*OsPSKR*5), AE-box (*OsPSKR*8 and15), ATCT motif (*OsPSKR*7), Box4(*OsPSKR*1, 3, 7, 9, 10, 13 and 14), Chs-CMA1a (*OsPSKR*8), CTAG motif (*OsPSKR*6,12 and 13), GAP- box (*OsPSKR*15), GATA-motif (*OsPSKR*3, 4, 5,8 and 10), GT1 motif (*OsPSKR*1,3,7,13 and 14), I-box (*OsPSKR*3, 4 and 11), Sp1 (*OsPSKR*10 and 14), TCCC motif (*OsPSKR*1, 6, 10, 12 and 15) and MRE (*OsPSKR*15).

The other category of motifs are generally involved in cellular development and are relatively less in number as compared to light, hormonal and abiotic stress responsive elements. These cis-elements include AC-I (*OsPSKR*6 and 12) which is involved in xylem specific expression, GCN4 (*OsPSKR*2 and 3) involved in endosperm expression, O2-site (*OsPSKR*3, 4, 7 and 15) involved in zein metabolism and circadian motif (*OsPSKR*8) has role in circadian control. Another cis-elements CAT box was present in all *OsPSKR*s except 5, 9, 10 and 13 and has role in meristem-specific activation.

To further improve our understanding for all identified *OsPSKR* genes, we looked at transcription factors targeting these *OsPSKR* genes. A list of transcription factors along with their *OsPSKR* targets is given in S3 Table. WRKY is the main transcription factor followed by AP2, MYB, dof, bZIP, homeobox, ARF18, ethylene responsive, zinc finger homeobox and myb like and more other transcription factors. Based on transcription factor target analysis, it can be hypothesized that PSKRs are involved in processes ranging from development (bZIPs, Zn Finger nucleases), flowering (GAGA, AP2) and stress responses (WRKY, MYB).

### Temporal and spatial expression analysis of PSKR genes in rice seedlings under abiotic stress

**Fig 9:**
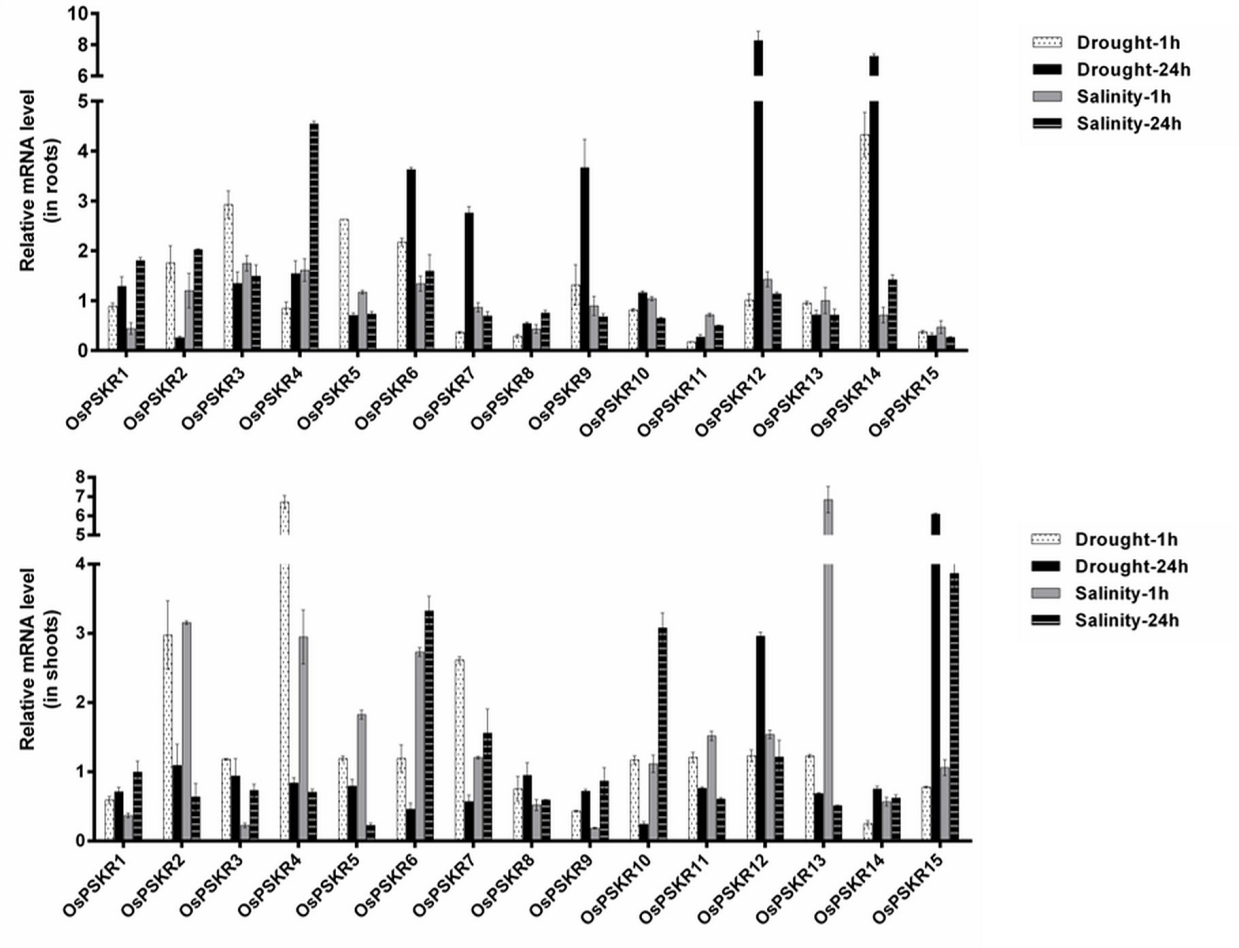
Relative expression analysis of fifteen *OsPSKR* genes under abiotic stress conditions. RT-PCR was used to analyze the expression patterns of the fifteen *OsPSKR* genes in root and shoot tissue of rice seedlings under high saline (200 mM NaCl) and drought conditions for 1 h and 24 h. The data were normalized to the rice *β-tubulin* expression level.

The relative transcript level of all the OsPSKRs was determined in response to salinity and drought stress in shoot and root tissue of rice seedlings at different time points. The real-time data reveals differential tissue specific stress inducibility of the members of PSKR gene family in *O*. *sativa* var. IR64 as shown in Fig 9.

Under abiotic stress condition, most of the *OsPSKR* genes were expressed in root tissue samples of indica rice var. IR-64. *OsPSKR*10, 11, 13 and 15 were exclusively expressed in shoot tissue while *OsPSKR* 2, 4, 5, 6, 7 and 12 were expressed both in root and shoot tissues. *OsPSKR*1, 3, 8, 9 and 14 did not change their expression in shoot tissue under abiotic stress condition.

*OsPSKR*s in the root tissue sample seem more responsive towards drought stress than the salinity stress. The expression of *OsPSKR*s like *OsPSKR*2, 3, 5, 6 and 14 was increased at 1 h time point in root tissue under water deficit condition, where *OsPSKR*3, *OsPSKR*5, *OsPSKR*6 and *OsPSKR*14 showed 3, 2.6, 2 and 4.5 fold increase in transcript level, respectively. *OsPSKR*6 (4.2 fold), *OsPSKR*7 (2.7 fold), *OsPSKR*9 (3.9 fold), *OsPSKR*12 (8.2 fold) and *OsPSKR*14 (7.2 fold) were late response members of this family in the same tissue under same stress. It was observed that in root tissue, *OsPSKR*6 and 14 are responsive to drought stress at both time points. Consistent with the RNA-seq results (Fig 6A), *OsPSKR* 12 showed significant upregulation under prolonged water deficit condition.

As seen in the Fig 9, the expression of OsPSKR*2* and *OsPSKR*4 enhanced by about 2 fold and 5 fold as late response to salinity stress. RNA-seq results from ssp. indica also showed the upregulation of *OsPSKR*2 under saline condition (Fig 6B), while the promoter analysis of *OsPSKR*4 depicted the presence of various abiotic stress responsive motifs (Fig 8).

In shoot tissue, the expression of most of the genes get upregulated except *OsPSKR*1, 3, 8, 9 and 14 in both stresses. Under drought condition, the expression of *OsPSKR*2, *OsPSKR*4 and *OsPSKR*7 increased by 2.9, 6.7 and 2.6 fold, respectively, at 1 h while in the same tissue, *OsPSKR*12 (2.9 fold) and *OsPSKR*15 (6 fold) showed the abundance in their transcript level in late hour. *OsPSKR*s expression also changed under salinity stress conditions. Most of them upregulated under 1 h saline conditions while *OsPSKR*6, 7, 10 and 15 were upregulated at late hour. The expression data shows that transcript level of *OsPSKR*2 increased by 3.1 fold while *OsPSKR*4 by 2.9, *OsPSKR*6 by 2.7 and *OsPSKR*13 by 6.8 fold as early event response, whereas *OsPSKR*6 (3.3 fold), *OsPSKR*10 (3 fold) and *OsPSKR*15 (3.8 fold) were upregulated in the shoot tissue in response to salinity stress at 24 h time point. The real time based expression analysis revealed *OsPSKR*2, 4, 6, 10, 12, 14 and 15 as important stress responsive candidate genes.

## Discussion

Yield losses in economically important crops is attributed to abiotic stress in multiple regions throughout the world. Increasing global temperatures because of global warming, would lead to crops being exposed to high temperatures, drought and saline soil. PSKRs are among the important LRR-RLKs that interact with other proteins including other LRR-RLKs to initiate a cascade of downstream signalling and play pivotal roles in different physiological processes as well as stress responses. However, there is little relevant information of this gene family from the most-important staple crop, *O*. *sativa* and also its role in abiotic stress signalling is yet to be explored. In the present study, we have identified fifteen members of PSKR gene family in *O*. *sativa* and named them according to their position on chromosome (Table 1). There are four tandemly duplicated gene pairs, three of these are located on chromosome 2 and one on chromosome 6. There are some differences between the tandem pairs, for example *OsPSKR*2 and *OsPSKR*3 are tandem duplicates but there are domain architecture differences between the two, OsPSKR2 had an extra LRR_4 domain (Fig 1). Duplication events including tandem and segmental duplications are very common in plants where tandem duplications are the most common ones. However in plants, duplications are accompanied by preferential retention of selected duplicates. Estimates of half-lives of duplicates suggest that changes occur very quickly in duplicates and some are lost in the process [63]. The high rate of duplications along with high rate of sequence changes could be the reason why some tandem duplicates vary significantly. Gene duplication can help plants to adapt to different environments during their development and growth [64]. Phylogenetic relationship between OsPSKRs and their orthologs (Fig 1A) revealed that there is dissimilarity between the PSKRs of monocots and dicots which leads to separate clustering of PSKRs depending on the plant species. These clusters of monocot and dicot PSKRs can further be divided into two more clades based on the differences in density of LRR domains.

Consistently with previous study conducted in *Arabidopsis* [20], rice PSKR proteins were mainly predicted to be localized to plasma membrane (Table 1). The presence of single transmembrane helix in the protein structure of all the members of PSKR family in rice suggested that these OsPSKRs are single pass transmembrane proteins (S2 Fig). The secondary structure prediction also showed the presence of transmembrane region (S2 Table). A typical PSKR contains an ectodomain with LRR repeats having island domain to interact with PSK ligand as well as co-receptor, SERK. Homology modelling and multiple sequence alignment of the ectodomain predicted that OsPSKR1, OsPSKR11 and OsPSKR15 were more identical to that of AtPSKR1/2 and DcPSKR than the rest of the members (Fig 2 and S4 Fig). Thus, unlike reported PSKRs, these identified OsPSKRs may perform in a similar way in terms of its stabilization and activation [19]. Besides the transmembrane region and LRR domain, the organization of kinase domain was also analysed. As indicated in Fig 3, similar to its orthologs (DcPSKR and AtPSKR1/2), all the members of OsPSKR family, showed the presence of conserved subdomains along with ATP binding site, CaM binding site, activation segment and guanylate cyclase centre in their cytoplasmic domains [19, 50]. *OsPSKR*9 expression is correlated with CaM binding protein which suggests its possible involvement in various developmental processes and adaptation responses through Ca^2+^ signalling (Fig 7).

Tissue specific expression pattern analysis provided valuable clues about the important roles of *OsPSKR* genes in rice growth, development and stress response. Expression profiles of fifteen *OsPSKR* genes were recorded in different tissues of rice (Fig 4). The results showed that most of the members were exclusively expressed in roots except *OsPSKR*2, *OsPSKR*8, *OsPSKR*14 and *OsPSKR*15 whereas *OsPSKR*8 and *OsPSKR*14 were most abundant in the leaf tissue indicating that they might play roles root development and leaf development, respectively. Previous studies have established the role of PSKR gene from *Arabidopsis* in growth and development [1, 14, 20, 21]. *OsPSKR2* and *OsPSKR*15 were abundant in panicle (Fig 4A) and embryo tissue (Fig 4B), indicating that they might be crucial for panicle development and embryogenesis. Interestingly, most of the other *OsPSKR*s including *OsPSKR*2 and *OsPSKR*15 (*OsPSKR*3, 6, 7, 8 and 14) transcripts were found to be increased in the rice plant embryo (Fig 4B). The role of PSK mediated signalling in pollen germination, fertilization and somatic embryogenesis has been already reported in a previous study [65]. *OsPSKR1*, *OsPSKR*4, *OsPSKR*9 and *OsPSKR*11 have higher transcript level in inflorescence and *OsPSKR*1 and *OsPSKR*13 in pistil which indicates their role in flower development and reproduction also [22, 23]. It has been observed (S3 Table) that these particular *OsPSKR*s are targeted by GAGA binding factors which are involved in flowering and AP2 domain binding proteins which are responsible for growth of plant, improvement and stress response [66, 67]. *OsPSKR*11 and 15 also have CAREs involved in abscisic acid signalling (Fig 8), so they might have role in embryo development and seed maturation [68].

Expression pattern profiles of different *OsPSKR*s for both ssp. japonica and indica were analyzed to get the better understanding of the putative role of OsPSKRs in biotic and abiotic stress signalling. Results provide insight into the possible role of *OsPSKR*6, *OsPSKR*8, *OsPSKR*10 and *OsPSKR*15 in initiating the response against bacterial leaf streak caused by *Xoc* in ssp. japonica (Fig 5A). A recent study has also reported the role of one of the identified OsPSKR in developing resistance to bacterial leaf streak in rice plant caused by RS105 strain of *Xoc* [27]. Some of the candidate genes like *OsPSKR*2, *OsPSKR*5, *OsPSKR*6, *OsPSKR*8, *OsPSKR*10, *OsPSKR*12 and *OsPSKR*15 showed their abundance in response to rice blast disease caused by *P*. *oryzae* (also known as *Magnaporthe oryzae*) in *O*. *sativa* ssp. indica (Fig 5B). The co-expression analysis showed the correlation of *OsPSKR*12 with NBS-LRR as well as Exo70 (Fig 7) and it has been reported that NBS-LRR recognize AVR-Pii, a small secreted protein of *M*. *oryzae*, through Exo70 to induce NLR-triggered immunity in plant against the fungal pathogen [53]. According to the heatmap (Fig 8), various important motifs including W-box are present in most of *OsPSKR*s as depicted by promoter analysis. WUN (*OsPSKR*1), H-box (*OsPSKR*5), Box S (*OsPSKR*8), and WRE3 (*OsPSKR*6, 8, 12 and 15) were also reported in *OsPSKR*s. The above results suggested the involvement of these OsPSKRs in regulating biotic stress signalling in rice.

To analyze the trend of the gene expression of *OsPSKR* genes under abiotic stress, we compared the results derived from qRT-PCR (Fig 9) and RNA-seq database (obtained for both ssp. japonica and indica) (Fig 6). The real time based expression analysis presented *OsPSKR*2, *OsPSKR*6, *OsPSKR*10, *OsPSKR*12 and *OsPSKR*15 as important candidate genes. Consistent with RNA-seq data of both ssp. japonica as well as indica, *OsPSKR*10 was upregulated under abiotic stress as shown by real time. Moreover, the promoter region of *OsPSKR*10 has most of the abiotic stress responsive motifs, for example, STRE, MYB-recognition site, asI, LTR and MYB like sequences (Fig 8). Transcription target analysis (S3 Table) also showed that *OsPSKR*10 also have target sites for transcription factors like WRKY36, WRKY37, WRKY45, WRKY67 and MYB family transcription factors which are important for regulation of stress response [56, 57]. Likewise, *OsPSKR*12 and *OsPSKR*15 were also upregulated in response to abiotic stress as depicted in expression analysis done by RNA-seq and real time data. The strong correlation between OsPSKR*12* and Exo70 (Fig 7) further supports the role of PSK-PSKR signalling in abiotic stress response as Exo70 is involved in stress tolerance besides its involvement in pollen maturation, germination and pollen tube growth and [54–56]. Moreover, the co-expression analysis of PSKR gene, as in Fig 7, showed that their expression is highly correlated to genes encoding for WRKY, MADS-box, NB-ARC, etc. whose functions are well established for abiotic and biotic stress responses [58, 59, 69].

In the present study, we predicted six types of hormone responsive *cis*-elements in the promoters of *OsPSKR* genes, including abscisic acid responsive, auxin-responsive, jasmonate-responsive, gibberellin-responsive, ethylene-responsive, and salicylic acid-responsive elements. Previous studies have already established that there is cross-talk between PSK signalling with various phytohormones in response to developmental and stress stimuli [4]. Studies have demonstrated the role of PSK signalling in modulating the salicylate- and jasmonate-dependent defense pathways [11], auxin-mediated pathways [25], brassinosteroids dependent growth process [9] and suppressing the ethylene (ET) production [28]. However, their involvement in ABA signalling is not well explored. ABA serves as central regulator of abiotic stress particularly drought stress responses in plants and its level increases in plant leaves. It coordinates a complex gene regulatory network enabling plant system to cope up with water deficit condition [70]. There are some other studies which showed that some members from LRR-RLK family, facilitate the cross-talk between abscisic acid and peptide signalling [71–73]. It has been observed that *OsPSKR*3, 7, 8 and 10, which are found to be upregulated under drought stress in either real time-PCR or RNA seq data, had motifs like ABRE, ABRE3a and ABRE3a in their promoter region (Fig 8). These results further supports our hypothesis that rice phytosulfokine receptor kinase family has role in development and stress signalling.

**Fig 10:**
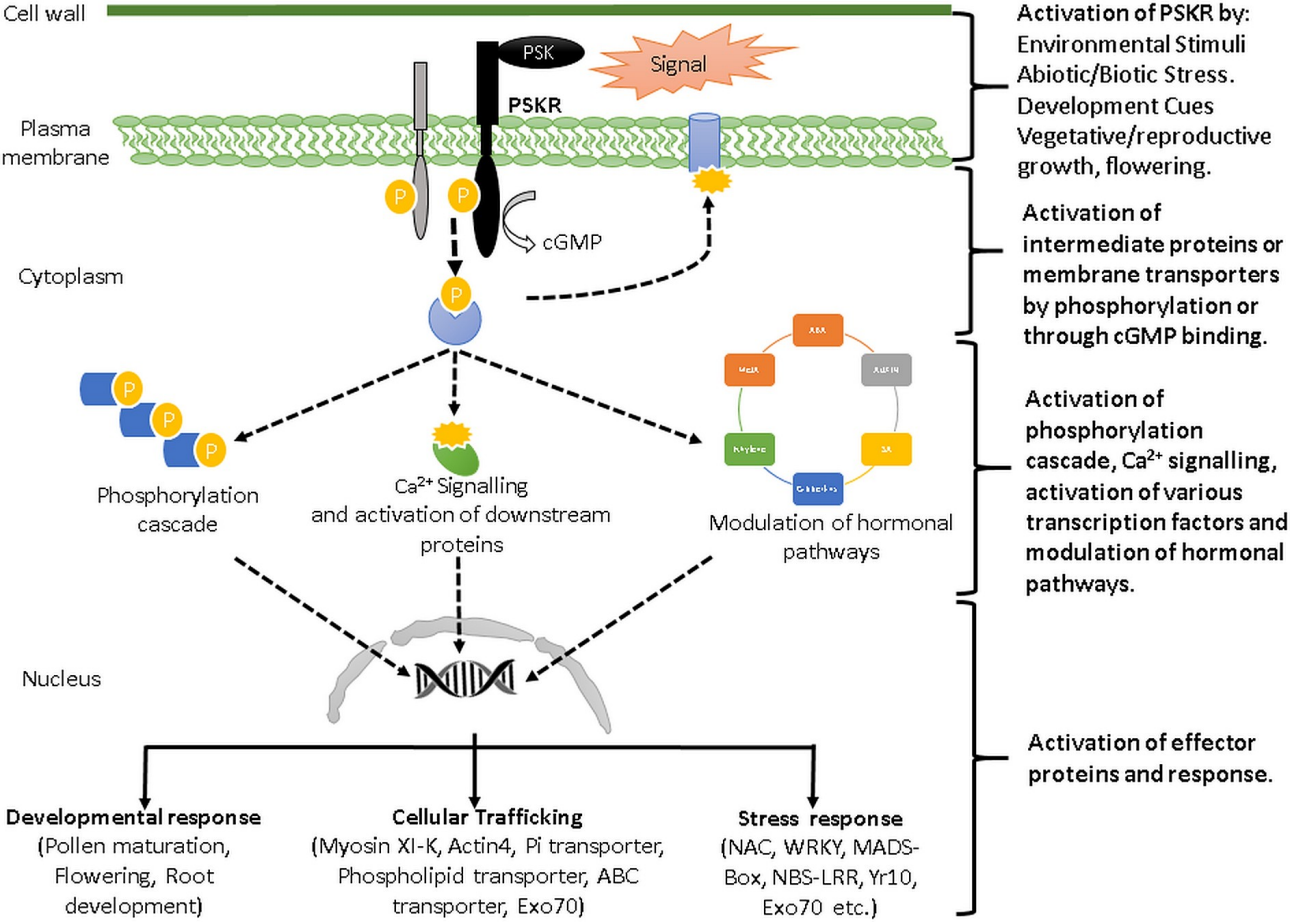
Hypothetical model of PSK-PSKR signalling based on environmental and developmental cues. PSKR gets activated on sensing the signal in the form of environmental or developmental cues [4, 27]. It leads to auto and cross phosphorylation of PSKR and other proteins [19]. The activated kinase domain of PSKRs further results in activation of other intermediate proteins or membrane transporters either by phosphorylation or cGMP binding [20]. This may initiate the phosphorylation cascade, Ca^2+^ signalling [17, 18, 25], activation of various transcription factors or modulation of hormonal pathways (Figs 7 and 8 and S3 Table) [9, 11, 25, 28]. In this way, these PSKR may further activate effector protein and generate stress response by regulating the expression of genes involved in developmental response, cellular trafficking and stress response (Figs 7 and 8) [1, 14, 20, 21]. Expression profiles based on RNA-seq (Figs 4–6) and real time (Fig 9) also suggested their role in development and stress signalling.

On the basis of the previous studies carried out on this gene family in other species and our present data, we have proposed a hypothetical model of PSK-PSKR mediated signalling (Fig 10). Our study will help researchers in understanding the role of phytosulfokine receptor mediated signalling during different stages of plant life span and also how it is involved in plant response to environmental cues.

## Conclusions

A total of fifteen genes in the PSKR family in *O*. *sativa* located on chromosome 2, 4, 6 and 7 of rice were identified. Gene structure analysis showed that these are evolutionary conserved genes and largely intronless. All OsPSKR proteins have extracellular LRR domain, transmembrane domain and kinase domain with ATP binding site, calmodulin-binding site, activation segment and guanylate cyclase centre. Transcription factor targeting, promoter element identification and co-expression analysis allowed us to predict the involvement of certain *OsPSKR* genes in various processes ranging from growth and development to stress response directly or indirectly. Results also indicated that there is cross talk between PSK-PSKR signalling and hormonal signalling. RNA-seq based expression pattern analysis suggested the involvement of *OsPSKR* genes in response to both biotic and abiotic stresses. RT-PCR based expression analysis further confirmed the strong response of *OsPSKR* genes, especially to drought but some show significant response to salinity stress also. Finally, we have identified probable stress responsive candidate genes like *OsPSKR*2, 10, 11, 12, 14 and 15 for further functional analysis.

## Supporting information

S1 Fig Chromosomal localization and gene duplication in *O*. *sativa* ssp. japonica. Distribution of *OsPSKR* genes on the different chromosomes of *Oryza sativa* ssp. japonica. Bars represent chromosome coordinates of *OsPSKR* genes in Megabase pairs. Colored pair of genes mark tandem duplication events. Genes connected by curve are segmental duplicates.
(TIF)

S2 Fig Transmembrane helices prediction for the PSKR proteins in rice by Phobius, a combined transmembrane protein topology and signal peptide predictor (http://phobius.sbc.su.se/). Signal peptide, transmembrane, cytoplasmic and non-cytoplasmic regions are represented in the chart. The plot shows the posterior probabilities of cytoplasmic, non-cytoplasmic, TM and helix/signal peptide.
(TIF)

S3 Fig Predicted 3D models of rice PSKR proteins. Models were generated by using Phyre2 server. Models were visualized by rainbow color from N to C terminus. Ten templates (c4y93A, c6s6qB, c2j0kB, c1oplA, c4mnaA, c4xi2A, c1y57A, c5gr8A, c4mn8A and c2fo0A) were used in the modelling of rice PSKRs, showing LRR domain at N terminus and transmembrane region followed by cytoplasmic kinase domain at C terminus.
(TIF)

S4 Fig Sequence alignment of the ectodomains of OsPSKRs with carrot DcPSKR and Arabidopsis AtPSKR1/2. Conserved and similar residues are boxed with red ground and red font, respectively. Based on the study [19], residues involved in recognition of PSK and interaction with a SERK member are indicated with blue solid circles and crosses at the bottom, respectively.
(TIF)

S1 Table List of *O*. *sativa* PSKR gene specific primers used for the real time PCR. (DOCX)

S2 Table Secondary structure prediction of OsPSKRs using Phyre2. Details of secondary structures like α-helix, β-strands and Transmembrane (TM) helices present in fifteen OsPSKRs were given in percentage (%).
(DOCX)

S3 Table Transcription factors targeting the *OsPSKR* genes. (XLSX)
