# Supplementary figures and images for: Genome-wide analysis and transcript profiling of PSKR gene family members in Oryza sativa
Source: PLoS One. 2020 Jul 23;15(7):e0236349. doi: 10.1371/journal.pone.0236349 (PMC7377467; doi:10.1371/journal.pone.0236349)

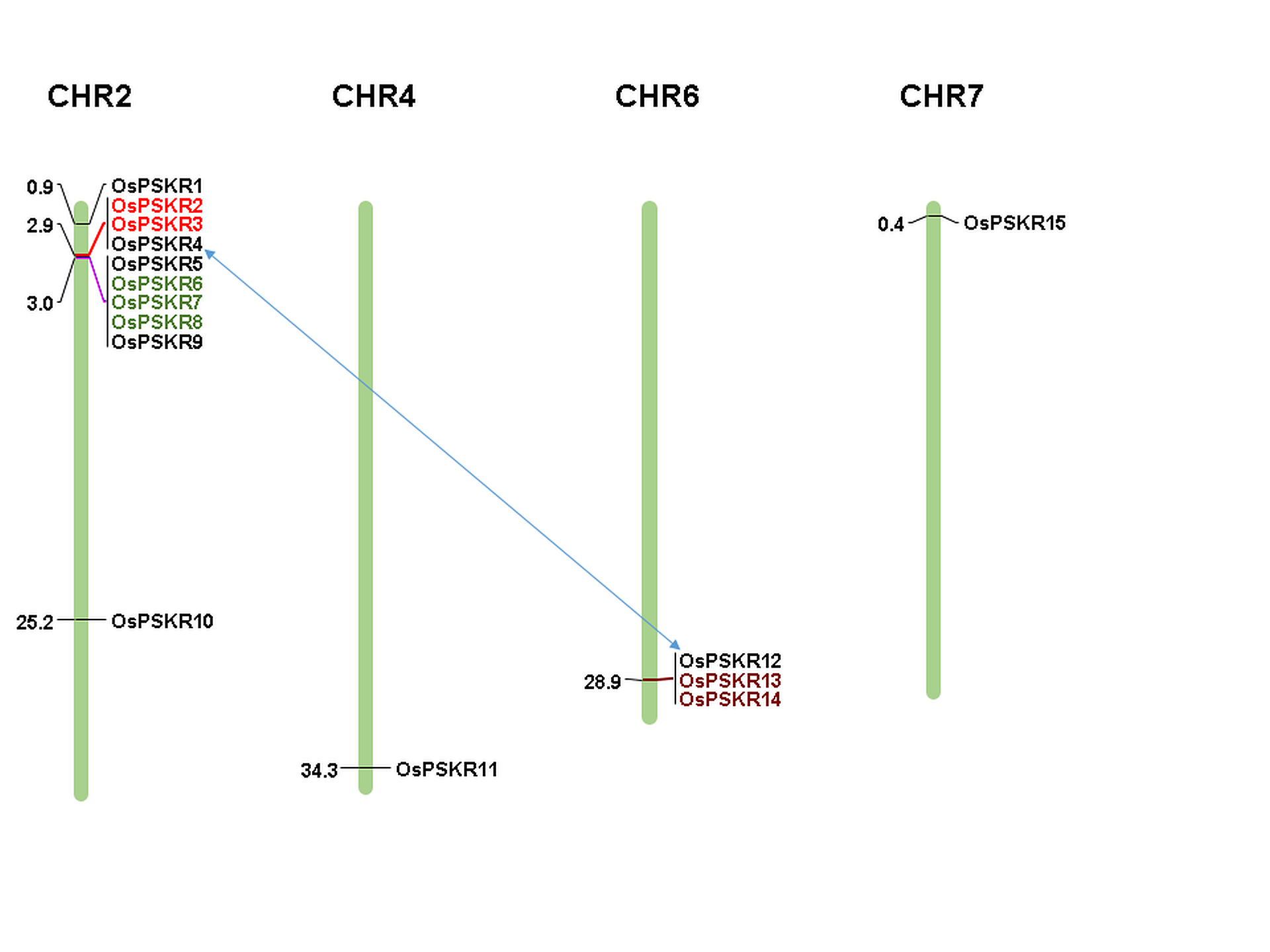

Supplement: S1 Fig — Distribution of OsPSKR genes on the different chromosomes of Oryza sativa ssp. japonica. Bars represent chromosome coordinates of OsPSKR genes in Megabase pairs. Colored pair of genes mark tandem duplication events. Genes connected by curve are segmental duplicates. (TIF) [file pone.0236349.s001.tif]

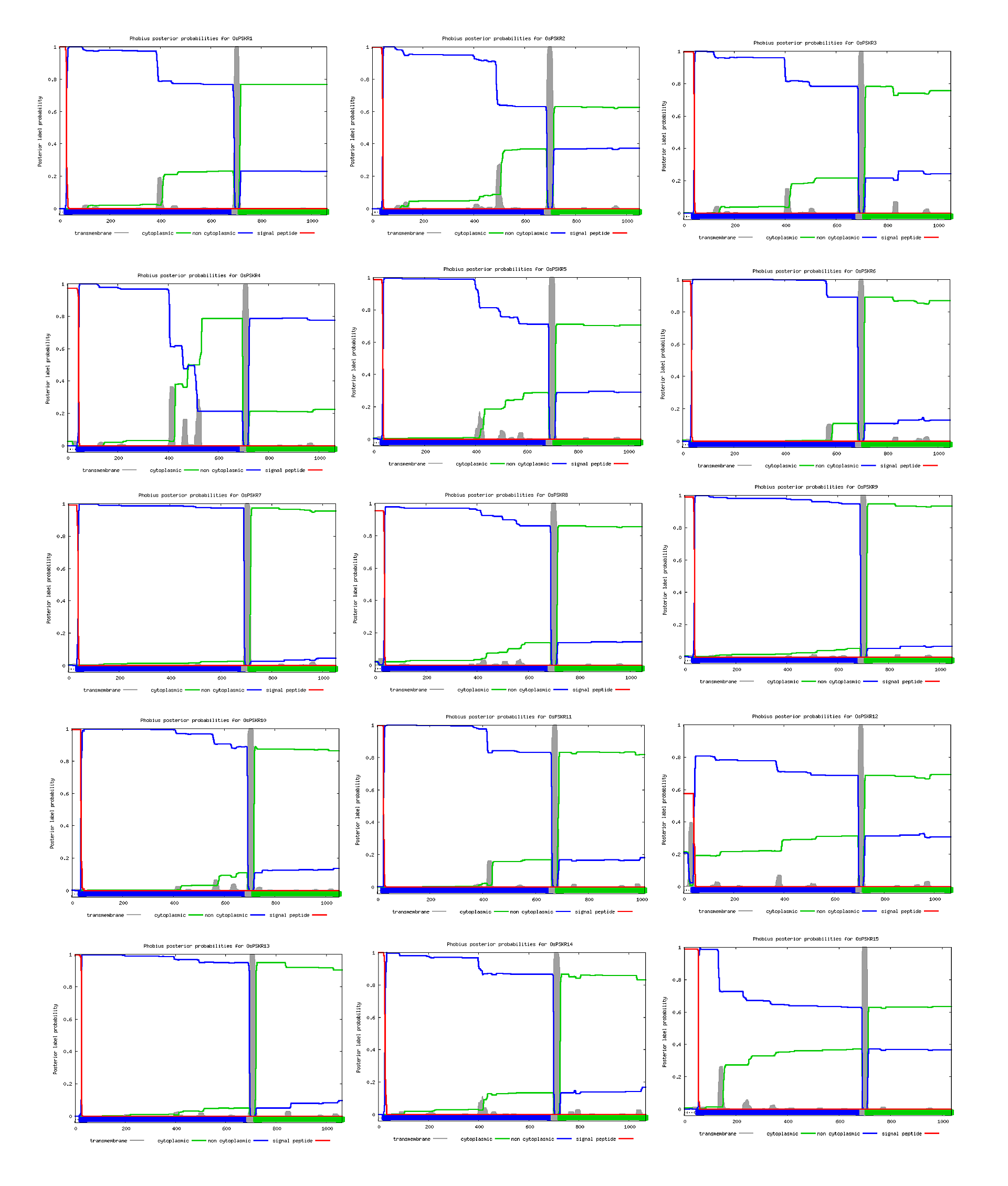

Supplement: S2 Fig — Signal peptide, transmembrane, cytoplasmic and non-cytoplasmic regions are represented in the chart. The plot shows the posterior probabilities of cytoplasmic, non-cytoplasmic, TM and helix/signal peptide. (TIF) [file pone.0236349.s002.tif]

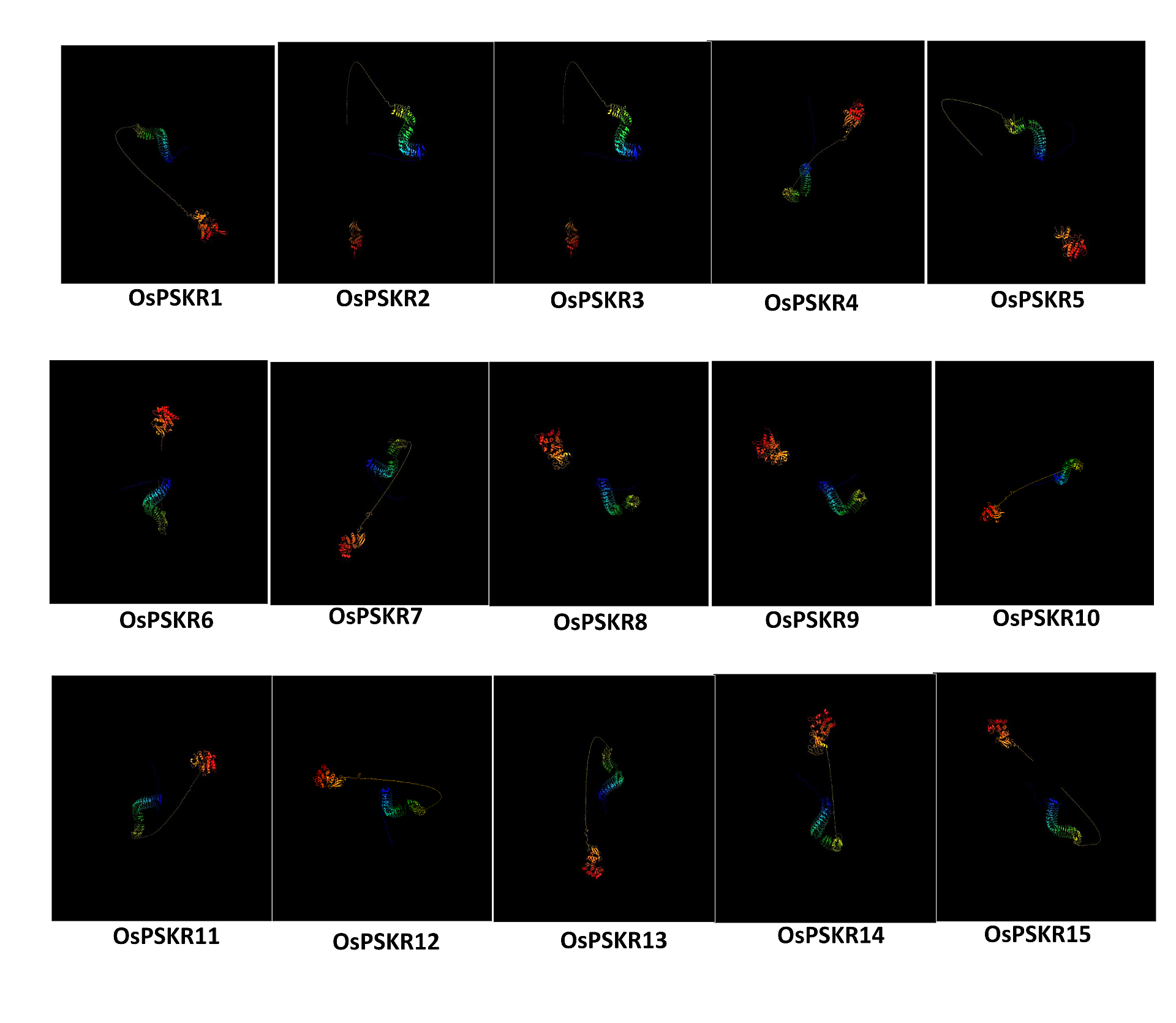

Supplement: S3 Fig — Models were generated by using Phyre2 server. Models were visualized by rainbow color from N to C terminus. Ten templates (c4y93A, c6s6qB, c2j0kB, c1oplA, c4mnaA, c4xi2A, c1y57A, c5gr8A, c4mn8A and c2fo0A) were used in the modelling of rice PSKRs, showing LRR domain at N terminus and transmembrane region followed by cytoplasmic kinase domain at C terminus. (TIF) [file pone.0236349.s003.tif]

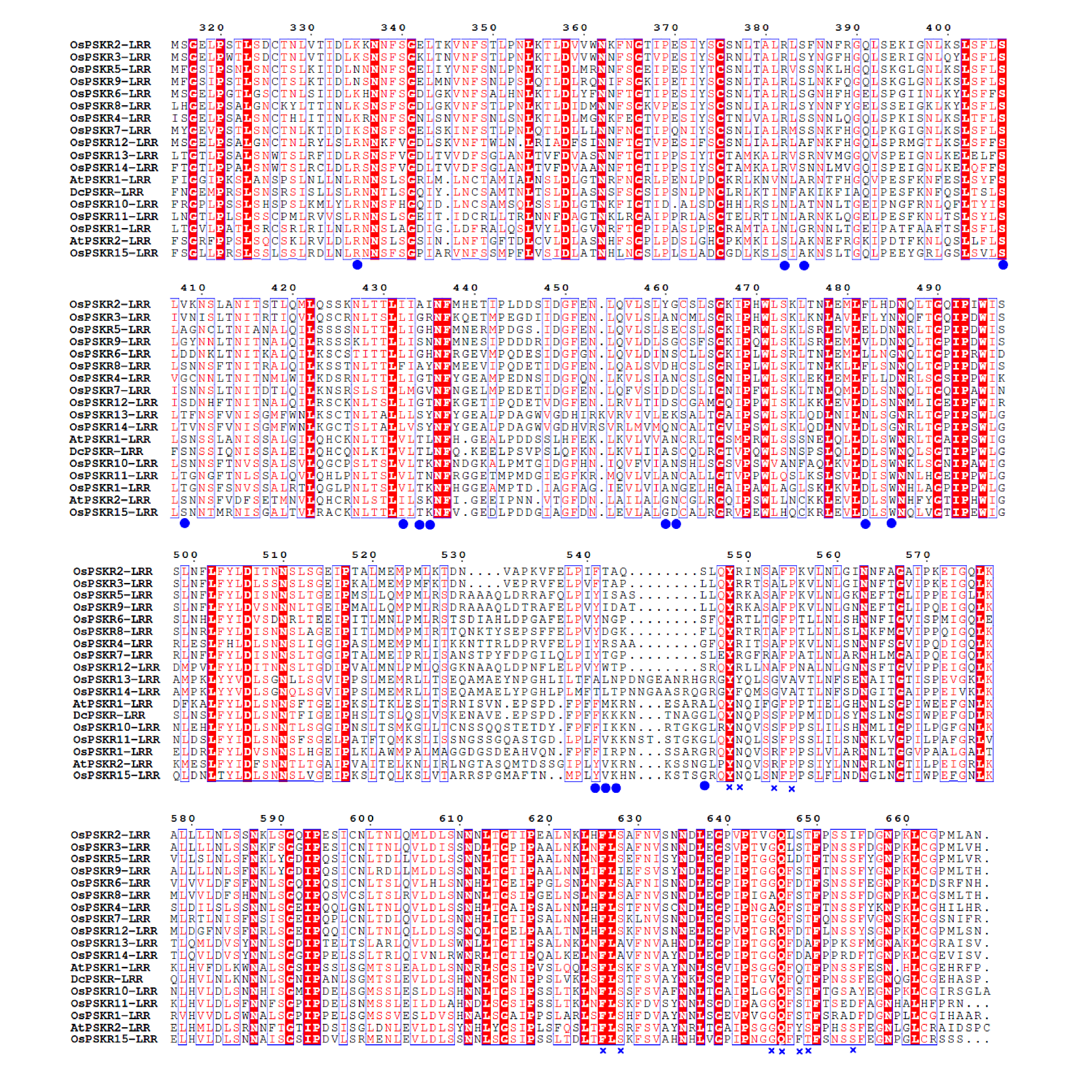

Supplement: S4 Fig — Conserved and similar residues are boxed with red ground and red font, respectively. Based on the study [19], residues involved in recognition of PSK and interaction with a SERK member are indicated with blue solid circles and crosses at the bottom, respectively. (TIF) [file pone.0236349.s004.tif]
