# Supplementary material for: Genome-wide analysis and transcript profiling of PSKR gene family members in Oryza sativa
Source: PLoS One. 2020 Jul 23;15(7):e0236349. doi: 10.1371/journal.pone.0236349 (PMC7377467; doi:10.1371/journal.pone.0236349)
Supplement: S1 Table — (DOCX) [file pone.0236349.s005.docx]

**S1 Table. List of *O. sativa* PSKR gene specific primers used for the real time PCR.**

| **S.No.** | **Primer Name** | **Sequence** |
| --- | --- | --- |
| 1 | PSKR1_real_F | GCAGCGTTCGAGTGAATCAG |
| 2 | PSKR1_real_R | GCCAAATCAGCAGTAATCCCAG |
| 3 | PSKR2_real_F | CTTCCTTTTTGCACAGTCCTCC |
| 4 | PSKR2_real_R | ATCACAGAATAATTTGCTGAACGA |
| 5 | PSKR3_real_F | ACCGAGCTGCAGACAACAAA |
| 6 | PSKR3_real_R | ACAGTGCAACACTCAGCTTCT |
| 7 | PSKR4_real_F | GTCCTAAGAGGTTATGAACACACA |
| 8 | PSKR4_real_R | TCCTGAAAACTAACCCACCTCG |
| 9 | PSKR5_real_F | CGTGTGACAGGCTAGTGTGAT |
| 10 | PSKR5_real_R | CGTGTGACAGGCTAGTGTGAT |
| 11 | PSKR6_real_F | AGGGAAAGCAGATTGAGGTGT |
| 12 | PSKR6_real_R | ACTTGCAAGCAGTTTCGAGC |
| 13 | PSKR7_real_F | CTGCCTGCAAGTGTGTCAAC |
| 14 | PSKR7_real_R | ATGTGTTTCGGTCAGCGTCT |
| 15 | PSKR8_real_F | TGCGGCCAACTATCACAGAG |
| 16 | PSKR8_real_R | CTTGACTGCCTTGTGCCTTC |
| 17 | PSKR9_real_F | GCAAGTGTGTCAAAGGTGATCC |
| 18 | PSKR9_real_R | ATCTTTAGGTCAGGCCGTCAG |
| 19 | PSKR10_real_F | CTCGGGAGTTGGTCTCATGG |
| 20 | PSKR10_real_R | GCACGGTCCAATACTTCAGC |
| 21 | PSKR11_real_F | TCAGCTACTGCTATGGTATGAATG |
| 22 | PSKR11_real_R | GCACTAGCTGGAGCAAATCAC |
| 23 | PSKR12_real_F | TGCAAGCCTCACATTGTTCAC |
| 24 | PSKR12_real_R | AAGGCCAAAATCCGCAACAC |
| 25 | PSKR13_real_F | GAGACGGCCCTTCGAGGT |
| 26 | PSKR13_real_R | CCTCATCTGGAGCACCCACTG |
| 27 | PSKR14_real_F | ACATGTCGGATTGTTCTGCCA |
| 28 | PSKR14_real_R | CAACTCACTGCTCCACCGAT |
| 29 | PSKR15_real_F | CTGAAACTTGGCCTTCCAGC |
| 30 | PSKR15_real_R | CACAAACCACCACTTATCTCCA |
