# Supplementary material for: Genome-wide analysis and transcript profiling of PSKR gene family members in Oryza sativa
Source: PLoS One. 2020 Jul 23;15(7):e0236349. doi: 10.1371/journal.pone.0236349 (PMC7377467; doi:10.1371/journal.pone.0236349)
Supplement: S2 Table — Details of secondary structures like α-helix, β-strands and Transmembrane (TM) helices present in fifteen OsPSKRs were given in percentage (%). (DOCX) [file pone.0236349.s006.docx]

**S2 Table. Secondary structure prediction of OsPSKRs using Phyre2.**

|  | **SECONDARY STRUCTURE PREDICTION (in %)** | | | | |
| --- | --- | --- | --- | --- | --- |
|  | **Coverage** | **Disordered** | **Alpha helix** | **Beta strand** | **TM helix** |
| **OsPSKR1** | 89 | 28 | 25 | 20 | 2 |
| **OsPSKR2** | 91 | 27 | 25 | 22 | 3 |
| **OsPSKR3** | 87 | 27 | 24 | 22 | 2 |
| **OsPSKR4** | 91 | 28 | 26 | 22 | 3 |
| **OsPSKR5** | 88 | 27 | 26 | 21 | 3 |
| **OsPSKR6** | 91 | 28 | 25 | 21 | 3 |
| **OsPSKR7** | 91 | 28 | 26 | 21 | 3 |
| **OsPSKR8** | 93 | 28 | 26 | 20 | 3 |
| **OsPSKR9** | 92 | 29 | 24 | 22 | 2 |
| **OsPSKR10** | 90 | 27 | 25 | 20 | 2 |
| **OsPSKR11** | 92 | 26 | 26 | 21 | 3 |
| **OsPSKR12** | 89 | 27 | 26 | 21 | 3 |
| **OsPSKR13** | 90 | 27 | 24 | 22 | 3 |
| **OsPSKR14** | 93 | 26 | 24 | 21 | 2 |
| **OsPSKR15** | 89 | 25 | 24 | 21 | 4 |
